# Genetic modification of *Clostridium kluyveri* for heterologous *n*-butanol and *n*-hexanol production

**DOI:** 10.1128/aem.00058-26

**Published:** 2026-03-13

**Authors:** Caroline Schlaiß, Saskia T. Baur, James W. Marsh, Kurt Gemeinhardt, Largus T. Angenent

**Affiliations:** 1 AG Angenent, Max Planck Institute for Biology 28329 , Tübingen, Germany; 2 Environmental Biotechnology Group, Department of Geosciences, University of Tübingen 9188, Tübingen, Germany; 3 Department of Microbiome Science, Max Planck Institute for Biology 28329 , Tübingen, Germany; 4 The Novo Nordisk Foundation CO2 Research Center (CORC), Aarhus University 1006, Aarhus, Denmark; 5 Department of Biological and Chemical Engineering, Aarhus University 1006, Aarhus, Denmark; 6 Cluster of Excellence – Controlling Microbes to Fight Infections, University of Tübingen 9188, Tübingen, Germany; University of Nebraska-Lincoln, Lincoln, Nebraska, USA

**Keywords:** *Clostridium kluyveri*, *n*-hexanol, metabolic engineering, chain elongation, *n*-caproate

## Abstract

The mesophilic microbe *Clostridium kluyveri* serves as the most commonly used model microbe to elucidate the physiology and biochemistry of ethanol-based chain elongation via reverse β-oxidation. In this pathway, ethanol and acetate are converted into short- and medium-chain carboxylates. However, to date, no genetic system has been published in a peer-reviewed publication. Here, we report the development of versatile genetic tools for *C. kluyveri*, utilizing the pMTL *Clostridia* shuttle vector system and thiamphenicol as a selective marker. We identified the native restriction-modification system of *C. kluyveri* as a critical barrier to DNA transfer and overcame it by identifying and characterizing the crucial methyltransferase. To mimic the native DNA methylation pattern of *C. kluyveri*, we performed *in-vivo* methylation of the shuttle vector plasmid by expressing the methyltransferase in *Escherichia coli*, followed by DNA transfer via conjugation. After validating the genetic system, we demonstrated heterologous expression of different combinations of both NADH- and NADPH-dependent alcohol dehydrogenases from *Clostridium acetobutylicum*. The expression of these genes was controlled by the P*_thl_* promoter, which is commonly used in *Clostridia*, and the P*_adhE2_* promoter, leading to *n*-butanol and *n*-hexanol production of the mutant strains. This genetic system for *C. kluyveri* will not only enable further research on the metabolism of this microbe but also enable more profound insights into ethanol-based chain elongation in general.

**IMPORTANCE:**

Medium-chain carboxylates are required in various everyday products, including cosmetics, pharmaceuticals, and fragrances, and show a natural antimicrobial property. Furthermore, they represent food additives and serve as chemical building blocks for several other compounds. Traditionally, these carboxylates are produced from fossil resources, contributing to increased greenhouse gas emissions. Alternatively, they are derived from animal- or plant-based fat (e.g., coconut oil), which competes with agricultural land that is needed for food production. However, microbial chain elongation, which is a biotechnological approach relying on microbes, such as *Clostridium kluyveri*, is sustainable and a promising alternative to the conventional production of medium-chain carboxylates. Notably, it enables the use of industrial waste streams (e.g., off-gases and carbohydrate-rich industrial waste) as substrates, making the process more environmentally friendly. By applying our genetic system for *C. kluyveri*, a better understanding of microbial chain elongation can be achieved and potentially even enable an extension of its product portfolio.

## INTRODUCTION

Despite advances in renewable energy technologies, modern society remains heavily dependent on fossil fuels. This continued reliance contributes considerably to greenhouse gas emissions, which in turn accelerate climate change and the depletion of natural resources (1). Therefore, moving toward a more circular economy represents a critical challenge for sustainability to ensure long-term resilience for future generations. A key aspect of this transformation is the development of sustainable alternatives for essential chemical building blocks, which are conventionally assembled using fossil resources.

The development of sustainable alternatives represents a central challenge for modern environmental biotechnology. In particular, chemical building blocks that are in high demand from industry include short-chain carboxylates (SCCs) and medium-chain carboxylates (MCCs) (2). Both SCCs and MCCs contain a carboxylic group. While SCCs have carbon chains with a length of 2–5 carbon atoms, MCCs consist of 6–12 carbons (3), and their monetary value increases with an increasing chain length (4). MCCs serve as versatile chemical compounds with numerous industrial applications. Beyond functioning as essential building blocks for bioplastics (5, 6) and potential biofuel precursors through Kolbe electrolysis (7–9), these compounds have considerable utility across multiple sectors. They are widely incorporated into cosmetics, pharmaceuticals (2), fragrances (10), and rubber production (11). Additionally, MCCs feature prominently in food products as flavor additives (11, 12) and in animal-feed formulations, where they provide valuable antimicrobial properties (13, 14). Also, they serve as precursors for alcohols such as *n*-butanol and *n*-hexanol (15).

Conventionally, MCCs are produced from petrochemicals (16), leading to environmental devastation and mobilization and release of carbon dioxide (CO_2_). A potential bioalternative that is already applied on an industrial scale is the production of MCCs from animal or plant fat, for example, coconut oil, palm kernel oil, and castor oil (17, 18). However, the sustainability of this approach is contested because it requires agricultural land, which is indispensable for growing food, especially given the steadily increasing global population (19). Another promising biotechnological alternative is microbial chain elongation to produce MCCs.

In the ethanol-driven reverse β-oxidation pathway, microbes fuse a C2 compound to short-chain carboxylates, resulting in a stepwise elongation of the carbon chain by two carbon atoms at a time, respectively, up to a maximum chain length of six carbon atoms (20). This elongation process can be achieved by reactor microbiomes using an open culture of microbial consortia. The open cultures do not require sterile treatment and are resilient to disturbances (21, 22). Another promising attempt, which was demonstrated in several studies, is to couple syngas fermentation with chain elongation by cultivating an acetogenic and a chain-elongating microbe in a coculture (23–26). First, the acetogenic microbe produces the intermediates ethanol and acetate from a mixture of hydrogen, CO_2_, and carbon monoxide. Second, the chain-elongating microbe further elongates the intermediates to medium-chain carboxylates via reverse β-oxidation. Third, the acetogenic microbe reduces SCCs and MCCs into their corresponding alcohols (*n*-butanol and *n*-hexanol). Besides open- and cocultures, medium-chain carboxylate production performed by pure cultures offers two to three times higher production rates and fewer by-products (27–30).

*Clostridium kluyveri* has been extensively used to study the physiology and biochemistry of ethanol-based chain elongation via the reverse β-oxidation pathway (31–34). Therefore, it serves as a model microbe for microbial chain elongation. To date, no genetic system for this microbe has been published in a peer-reviewed study. Here, we identified the restriction-modification system of *C. kluyveri* as the primary barrier to genetic modification. This system recognizes foreign DNA based on methylation patterns that differ from the native DNA methylation of the host and expresses restriction enzymes that cleave the unmethylated recognition sites (35). We developed a genetic system for *C. kluyveri* by mimicking its native methylation pattern on a shuttle vector plasmid to circumvent its defense system. As a proof of principle, we introduced and heterologously expressed alcohol dehydrogenases from *Clostridium acetobutylicum* to enable *n*-butanol and *n*-hexanol production. By applying the findings from this study, metabolic engineering of *C. kluyveri* can be used to increase its productivity, expand its product portfolio, and obtain more profound insights into the reverse β-oxidation metabolism in general.

## RESULTS

### Plating optimization

To enable successful genetic modification, our initial focus was to optimize plating conditions for the reliable growth of single colonies, because a high plating efficiency increases the likelihood of isolating the correct clone. We systematically adjusted each variable to determine the optimal plating conditions using duplicates. Of these, supplementing the DSMZ52 medium with a 10-fold concentration of vitamins and using cells in their early exponential growth phase (Fig. S1) had the most substantial positive impact on colony formation (Table S1). Other modifications, such as pour-plating with 0.8% Bacto agar, increasing the atmospheric pressure in the storage tanks to 0.6 bar, and placing a sterile filter paper (1 cm × 1 cm) soaked with ethanol in the lid of the Petri dish to ensure sufficient substrate supply, showed moderate positive effects (Table S1).

In contrast, using complex modified reinforced clostridial medium (RCM) (36), which was supplemented with 20 mL/L ethanol and 2.5 g/L NaHCO_3_ instead of DSMZ52 medium, and adding ethanol to the external plate atmosphere affected the plating efficiency negatively (Table S1). Furthermore, using paper clips to increase ventilation resulted in a drastic reduction in colony formation (Table S1), most likely due to ethanol evaporation and plate desiccation from the enhanced airflow. Because hydrogen production of *C. kluyveri* resulted in large bubbles of the pour-plated medium, which complicated post-treatment, we decided to perform spread-plating whenever a minor reduction in plating efficiency was acceptable. For routine plating, we chose DSMZ52 medium with increased vitamin supplementation over RCM to reduce contamination risk and improve consistency, resulting in a plating efficiency of 77.2% ± 14.1% (Table S2). However, RCM medium was necessary during conjugation to support the growth of *Escherichia coli*.

### Antibiotic testing

Selection of cells carrying the desired plasmid that confers a new antibiotic resistance requires two key factors: (i) the sensitivity of the wild-type strain to the respective antibiotic and (ii) the expression of a reporter, such as an enzyme, that ensures resistance to the antibiotic to which the parental strain is sensitive. Thiamphenicol resistance is a well-established marker in many clostridial strains, including strains closely related to *C. kluyveri* (37, 38). Therefore, we tested the sensitivity of the cells to thiamphenicol in liquid DSMZ52 medium and on plates by using duplicates and determined the minimum inhibitory concentration (MIC) after 1 week, which was 3 µg/mL (Fig. S2). We repeated the experiment once independently and obtained consistent results, which align with the literature on *Clostridia* (36, 38, 39). However, we chose to use a higher concentration of 5 µg/mL thiamphenicol in liquid medium to prevent the growth of wild-type cells due to spontaneous resistance or a reduction in antibiotic concentration caused by instability of the antibiotic at 37°C after prolonged incubation. Additionally, we used this concentration to select plasmid-carrying cells on solid media.

### Identification of the methylation motifs of genomic *C. kluyveri* DNA

**Fig 1:**
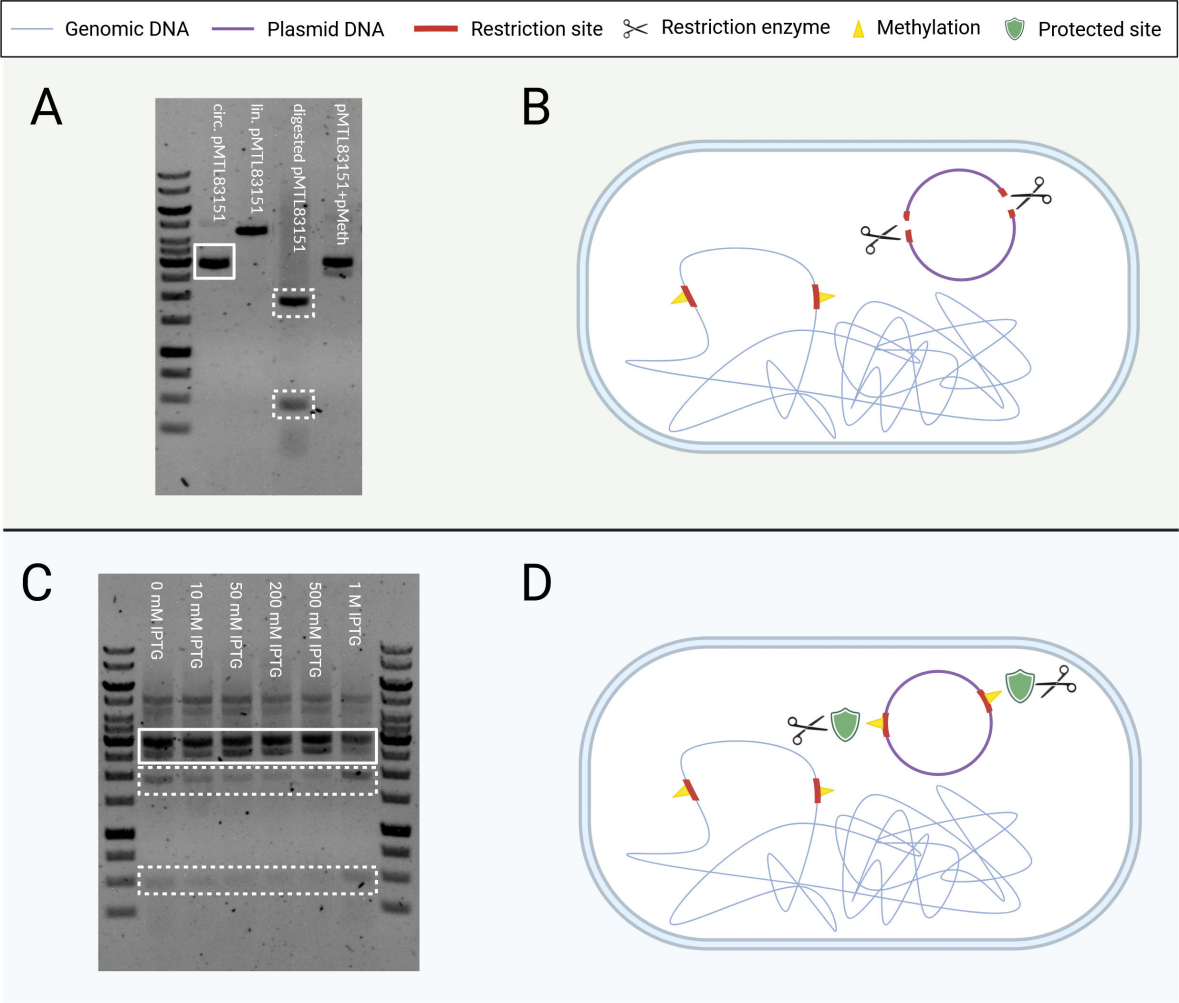
Restriction-modification system of *C. kluyveri*. (**A**) Restriction digest of the unmethylated pMTL83151 plasmid after 4 h. The solid-lined box in the second lane shows the unrestricted, circular plasmid (4.476 kbp) running at ~3 kbp due to its supercoiled conformation, whereas the dashed-lined box in the fourth lane shows the vector digested by the *C. kluyveri* cell lysate (fragment sizes: 1.899, 0.485, and 0.483 kbp, and some smaller fragments not detectable on the gel [see Table S5], visible on the gel as two bands at ~2 and ~0.5 kbp, respectively). The pMTL83151 vector was linearized with the restriction enzyme *Sbf*I and applied in the third lane (~4.5 kbp), and the mixture of circular plasmids pMTL83151 and pMeth (fifth lane) served as control. (**B**) In bacterial cells, DNA restriction serves as a defense mechanism against foreign DNA that either lacks methylation at the critical sites or shows a different methylation pattern from the native DNA of the respective bacterium. (**C**) Restriction digest of *C. kluyveri*-specific methylated pMTL83151 after 24 h. Because the expression of the methyltransferase (CKL_2671) of *C. kluyveri* was controlled by the P*_lac_* promoter, we tested different IPTG levels for induction (from 10 µM to 1 mM). All induction levels result in minor restriction of pMTL83151 highlighted with dashed boxes (fragment sizes: ~2 and ~0.5 kbp); however, the restriction was clearly reduced and minimized. Best results with the least restriction were obtained for the induction with 500 µM IPTG. This experiment was performed once. (**D**) As a result of the same methylation pattern as the native DNA, the foreign DNA is protected from restriction by the bacterial restriction-modification system. Created with BioRender.com.

A restriction assay of the pMTL83151 shuttle vector plasmid (40), using the cell lysate of *C. kluyveri*, showed digestion of the foreign DNA, producing fragment sizes of approximately 2 and 0.5 kbp (Fig. 1A). This result indicated that the native restriction and modification defense system of *C. kluyveri* (Fig. 1B) is a critical barrier that must be overcome for successful genetic modification. PacBio sequencing (complete data of the analysis of the native methylation pattern are shown in Table S4 and S5) of the native methylation pattern of the genomic DNA of *C. kluyveri* DSM555 revealed methylation at the following DNA sites: GATC, GWTAAT, CCAAG, CAAAAAT, and CCGG, suggesting the presence of multiple restriction-modification systems. *In-silico* analysis revealed that the observed restriction pattern of the unmethylated pMTL83151 corresponded to the CCGG as the critical methylation (Table S5), corresponding with the findings of Schneider (41).

We identified a native methyltransferase in the REBASE database (42) that methylates CCGG sites and is encoded by the genome of *C. kluyveri* (locus tag CKL_2671). To counteract the restriction, we expressed this methyltransferase under the control of the isopropyl-β-D-thiogalactopyranoside (IPTG)-inducible P*_lac_* promoter on the plasmid pMeth (Fig. S3) to mimic *C. kluyveri*-specific methylation of pMTL83151. As a result, the restriction assay testing CCGG-methylated pMTL83151 showed reduced plasmid digestion. Next, we optimized the methyltransferase expression by testing different IPTG concentrations (100–1,000 µM) for induction in *E. coli* TOP10. In a final restriction digest (Fig. 1C), we observed only minimal digestion of the shuttle vector after 24 h, indicating that the restriction sites of the foreign DNA were protected by methylation (Fig. 1D). The lowest restriction activity occurred at IPTG induction concentrations between 200 and 500 µM right after inoculation. Based on these results, we established 500 µM IPTG as the standard concentration for inducing the expression of the methyltransferase.

### Triparental conjugational DNA transfer

The *E. coli* TOP10 strain, harboring both pMTL83151 and pMeth plasmids, exhibited reduced growth rates. This growth inhibition was likely attributable to the metabolic burden that was imposed by maintaining resistance to two antibiotics simultaneously (chloramphenicol for pMTL83151 and ampicillin for pMeth), combined with the additional cellular stress of protein expression that is induced by IPTG treatment. To avoid additional burden on this strain by introducing a third plasmid to enable conjugation, we instead performed triparental mating for shuttle vector transfer into the *C. kluyveri* recipient using a modified transformation protocol (43). We retained *E. coli* TOP10, containing both pMeth and the shuttle vector plasmid, as the donor strain and *E. coli* HB101, carrying pRK2013, as the helper strain (44). To support the growth of the *E. coli* strains and *C. kluyveri* on the same plate, we tested the growth of the two *E. coli* strains on RCM plates supplemented with varying ethanol concentrations (86–342 mM, 0.5%–2.0% [vol/vol]) under a N_2_/CO_2_ atmosphere (Fig. S4). Both *E. coli* strains exhibited normal growth up to 257 mM ethanol. We decided to use the highest ethanol concentration because it is the closest to the ethanol level that we use to cultivate *C. kluyveri* (342 mM), making it the optimum condition for cocultivating these three strains.

We evaluated different mating durations (24, 48, 72, 96, and 120 h) by selecting potentially transformed cells on plates containing thiamphenicol and trimethoprim. After 24 h, individual colonies were visible (Fig. S5). However, when extending mating times from 48 to 72 h, a higher number of CFUs on the selective plate containing antibiotics was observed. In the next step, we successfully verified the presence of the shuttle vector plasmid inside *C. kluyveri* cells via colony PCR (Fig. S6A), and we sequenced the 16S rRNA gene to verify pure cultures (Fig. S6B). Based on the calculated transformation efficiencies, mating for 24 h resulted in the highest mean value, whereas prolonged mating times of 48 h and 72 h yielded slightly lower but comparable efficiencies (Table S3). Because transformation efficiencies were normalized to the cell numbers, these values do not directly reflect the absolute number of CFUs obtained, yet plasmid transfer was robustly achieved at all tested mating times.

### Heterologous gene expression as proof of concept

Because the reverse β-oxidation pathway represents the central energy metabolism of *C. kluyveri*, directly targeting it carries the risk of causing lethal or unpredictable effects. Therefore, as a proof of principle, instead of interfering with its core metabolism, we decided to expand the product portfolio of *C. kluyveri* by enabling the conversion of butyryl-CoA and hexanoyl-CoA into *n*-butanol and *n*-hexanol. This was achieved by introducing the *adhE2* gene from *C. acetobutylicum* into the shuttle vector plasmid pMTL83151 under the control of its native promoter, which is P*_adhE2_. adhE2* encodes a bifunctional alcohol dehydrogenase, which catalyzes both the conversion of acyl-CoA to the corresponding aldehyde in the first step and the reduction of the aldehyde to the respective alcohol in the second step (45). After successful transformation of the plasmid into *C. kluyveri* cells, we observed the production of alcohols corresponding to the chain length of the carboxylates produced. However, preliminary measurements of the alcohols produced by *C. kluyveri* pPadhE2_adhE2 revealed relatively low alcohol concentrations (*n*-butanol: 1.87 mM, *n*-hexanol: 3.15 mM). To increase alcohol production, we tested two conditions: (i) increasing the expression of the *adhE2* gene by using a stronger promoter (46) and (ii) introducing an NADPH-dependent alcohol dehydrogenase because NADPH represents a more powerful cofactor than NADH (47), and the use of NADH by AdhE2 potentially lowers the NADH/NAD+ ratio too much. Therefore, using NADPH as an alternative cofactor could be advantageous for *n*-butanol and *n*-hexanol production.

**Fig 2:**
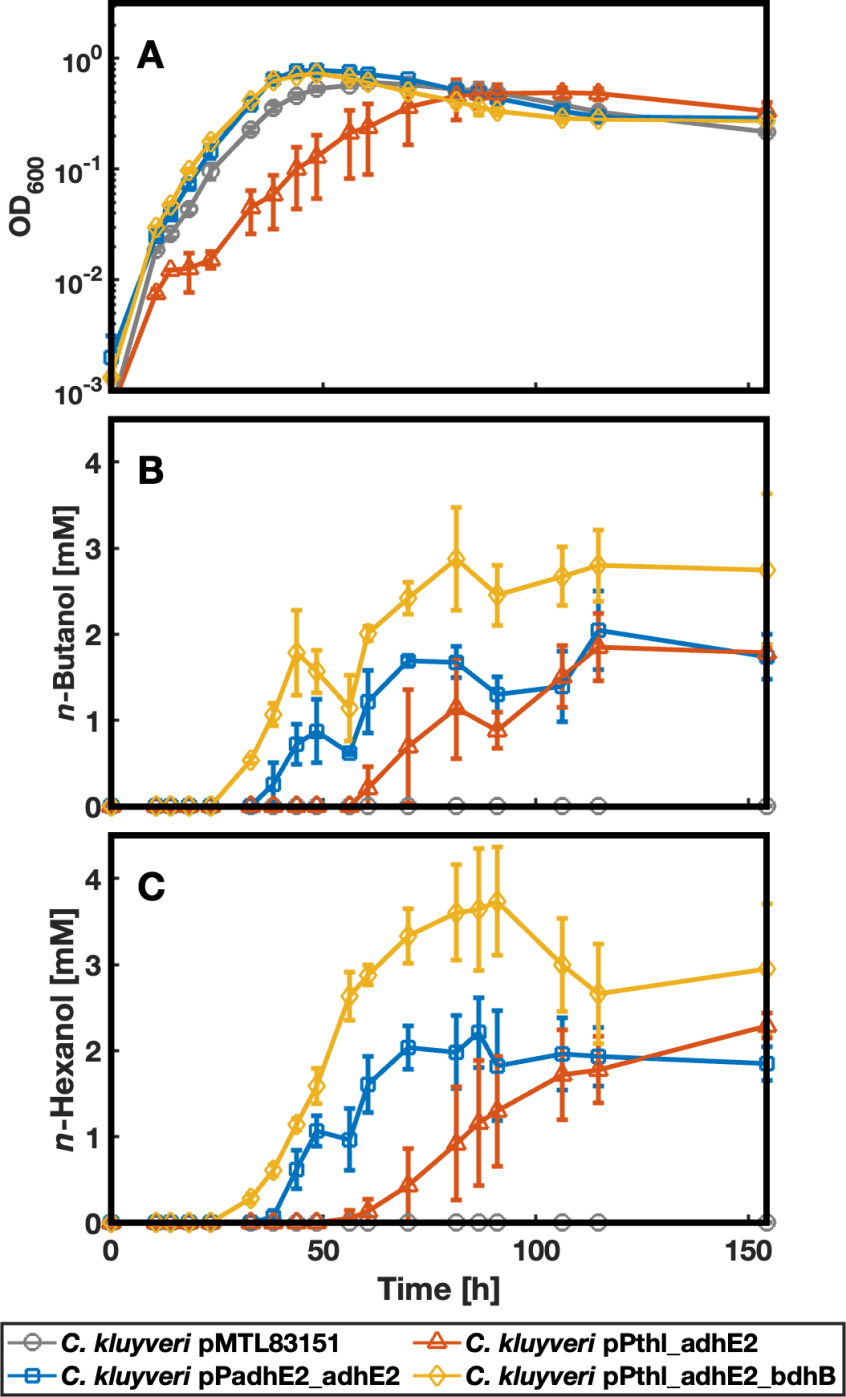
Growth and alcohol production of the engineered strains. We carried out three independent growth experiments. This figure shows data from the experiment in which all three biological replicates of *C. kluyveri* pPthl_adhE2 exhibited successful alcohol production. The results of the other two experiments, where promoter mutations affected the performance of *C. kluyveri* pPthl_adhE2, are presented in Fig. S8. The experiment was performed in biological triplicate, and the error bars represent standard deviations. (**A**) The growth behavior was measured by OD_600_, showing that all strains but *C. kluyveri* pPthl_adhE2 showed comparable growth. (**B**) *C. kluyveri* pPthl_adhE2_bdhB reached the highest *n*-butanol concentration, whereas *C. kluyveri* pPadhE2_adhE2 and *C. kluyveri* pPthl_adhE2 showed comparable production levels. (**C**) We observed the highest *n*-hexanol levels for *C. kluyveri* pPthl_adhE2_bdhB, followed by *C. kluyveri* pPthl_adhE2. Created with MATLAB 2024b.

To address these limitations, we enhanced *adhE2* expression by replacing the native promoter with the reportedly stronger P*_thl_* promoter from *C. acetobutylicum* (48, 49), which resulted in higher *n*-butanol and *n*-hexanol concentrations (*n*-butanol: 2.73 mM, *n*-hexanol: 3.25 mM) compared to the P*_adhE2_* promoter. To further increase alcohol production, we introduced the *bdhB* gene from *C. acetobutylicum*, encoding butanol dehydrogenase B. Unlike AdhE2, BdhB uses NADPH as a cofactor for converting aldehydes to alcohols (50, 51). Since BdhB exclusively catalyzes the second step from the aldehyde to the alcohol (52), we retained AdhE2 to ensure acyl-CoA conversion to the corresponding aldehyde. Thus, we tested three constructs on the shuttle vector plasmid pMTL83151: (i) pPadhE2_adhE2, (ii) pPthl_adhE2, and (iii) pPthl_adhE2_bdhB and used the pMTL83151 vector as the negative control in a growth experiment (Fig. 2). We successfully transformed *C. kluyveri* cells with the three constructs and the negative control and evaluated the resulting phenotypes. Notably, during the conjugation process, we observed that DNA transfer for the plasmid encoding both *adhE2* and *bdhB* was only successful after 72 h of mating.

### *n*-Butanol and *n*-hexanol production of the different strains

In batch cultivation, *C. kluyveri* pPadhE2_adhE2, *C. kluyveri* pPthl_adhE2_bdhB, and the negative control exhibited comparable growth with doubling times between 4.5 and 8 h (Fig. 2A; Table S6). In comparison, the three replicates of *C. kluyveri* pPthl_adhE2 started to grow at different times after inoculation, and thus leading to higher standard deviations (not only for the OD_600_ but also for substrate and product concentrations for this strain [Fig. 2; Fig. S7]). Unfortunately, two independent attempts to repeat the growth experiment failed (Fig. S8) due to a high mutation rate in the promoter region of *adhE2* in *C. kluyveri* pPthl_adhE2, leading to the strains’ inability to produce both *n*-butanol and *n*-hexanol (Fig. S9A). Additionally, Sanger sequencing of the P_*thl*_ promoter region from all three biological replicates of *C. kluyveri* pPthl_adhE2, in the experiment where this strain produced *n*-butanol and *n*-hexanol, revealed that a subset of the cells carried a mutation in this promoter region (Fig. S9B). Average *n*-butanol and *n*-hexanol production was first observed after 33 h for *C. kluyveri* pPthl_adhE2_bdhB and after 38 h for *C. kluyveri* pPadhE2_adhE2 (Fig. 2B and C). Due to the extended lag phase, *C. kluyveri* pPthl_adhE2 was the last strain starting to produce *n*-butanol and *n*-hexanol (first production observed between 61 and 81 h [Fig. 2; Fig. S7]).

**Fig 3:**
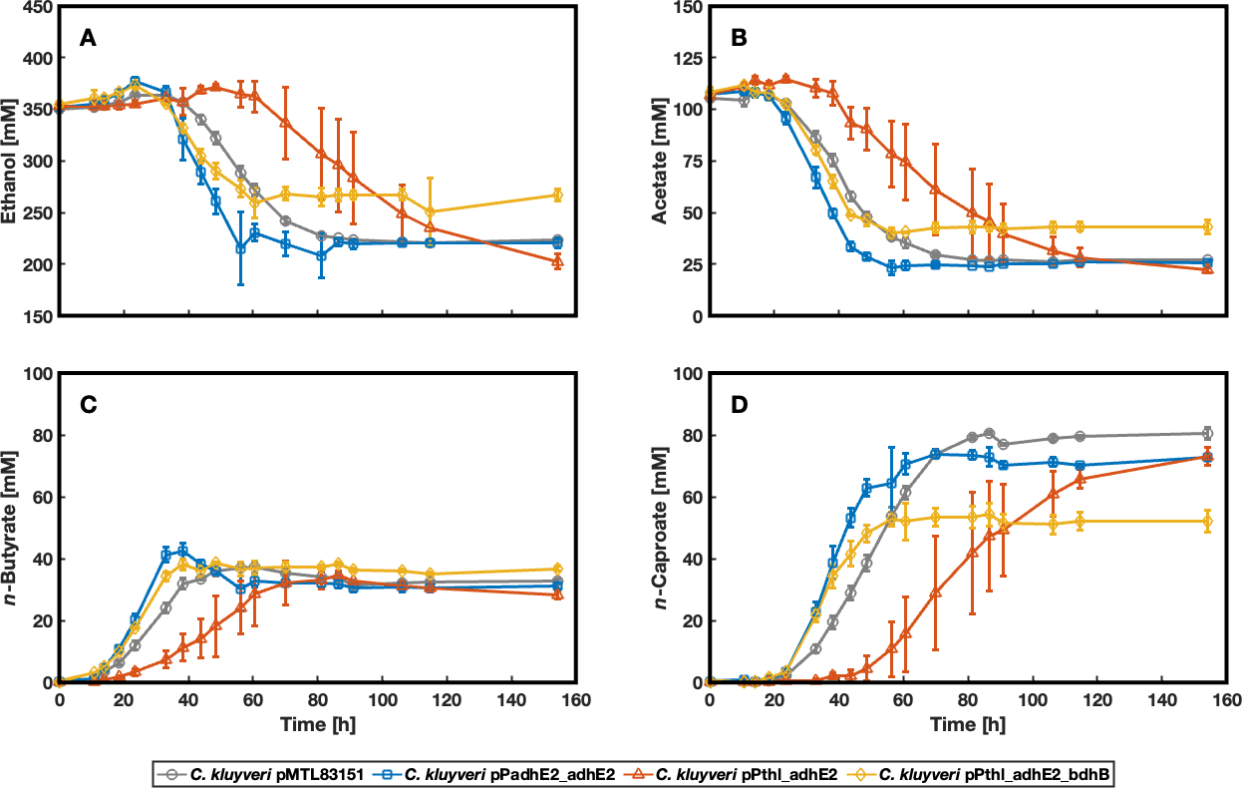
Concentrations of the substrates and the produced carboxylates from the engineered strains. The experiment was performed in biological triplicate, and the error bars represent standard deviations. (**A** and** B**) Ethanol and acetate are consumed by all strains; however, they are consumed more slowly by *C. kluyveri* pPthl_adhE2 than by the other strains due to its extended lag phase. *C. kluyveri* pPthl_adhE2_bdhB showed the lowest ethanol consumption at the end of the experiment. (**C**) All strains showed comparable *n*-butyrate production of approximately 35 mM, (**D**) while *C. kluyveri* pMTL83151 achieved the highest production of *n*-caproate. Corresponding to the lowest substrate consumption, *C. kluyveri* pPthl_adhE2_bdhB showed the lowest *n*-caproate concentration after 50 h. Created with MATLAB 2024b.

Regarding *n*-butanol production, *C. kluyveri* pPthl_adhE2_bdhB reached a maximum concentration of 4.0 ± 0.3 mM (Table S7), whereas *C. kluyveri* pPadhE2_adhE2 and *C. kluyveri* pPthl_adhE2 exhibited similar *n*-butanol production levels (2.2 ± 0.3 and 2.0 ± 0.3, respectively; Fig. 2B; Table S7). In line with this observation, *C. kluyveri* pPthl_adhE2_bdhB also achieved the highest *n*-hexanol concentration (3.9 ± 0.6 mM), followed by *C. kluyveri* pPthl_adhE2 (2.3 ± 0.1 mM). *C. kluyveri* pPadhE2_adhE2 produced the lowest amount of *n*-hexanol (2.2 ± 0.3 mM) (Fig. 2C; Table S8). Interestingly, both strains carrying P*_thl_* started producing the corresponding alcohols at lower *n*-butyrate and *n*-caproate concentrations (32.8 ± 2.7 mM and 21.01 ± 4.7 mM, and 34.3 ± 1.2 mM and 21.7 ± 1.8 mM, respectively) (Fig. 2B and C; Table S9 and S10). In contrast, *C. kluyveri* pPadhE2_adhE2 started *n*-butanol and *n*-hexanol production at 40.21 ± 1.2 mM *n*-butyrate and 42.6 ± 10 mM *n*-caproate (Fig. 2B and C, Fig. 3C and D; Table S9 and S10). Remarkably, *C. kluyveri* pPthl_adhE2_bdhB converted most acyl-CoA into the corresponding alcohol and reached the highest OD_600_ (Fig. 2A and B). It produced approximately 22% less *n*-caproate than the other alcohol-producing strains and approximately 30% less than the negative control (Fig. 3D; Table S12). Due to the lower *n*-caproate production, its conversion rate for substrate carbon into product carbon reached only 53%, whereas the other strains converted 62%–67% of it (Fig. 2B and C, Fig. 3; Table S13). Furthermore, *C. kluyveri* pPthl_adhE2_bdhB showed a lower ethanol-to-acetate ratio at the end of the experiment (6.2) than the other strains (8.2–9.2) (Fig. 3A and B; Table S14). The carbon recovery from the metabolized substrates was close to 100% in all strains (Table S15).

## DISCUSSION

Our results show that (i) adapting the growth conditions for *C. kluyveri* on solid DSMZ52 medium, (ii) determining the MIC of thiamphenicol, and (iii) counteracting restriction-modification barriers enabled successful DNA transfer of a shuttle vector plasmid into *C. kluyveri* cells via conjugation after 24 h of mating. Although extending the mating time to 48 h resulted in more CFUs, the overall transformation efficiency decreased after normalization to the growth of the recipient cells on the mating plate (Table S3). Notably, transferring the plasmid pPthl_adhE2_bdhB (8,234 kbp) was only successful after 72 h, suggesting that shuttle vectors with critical genes or an increased size may require extended mating times.

Although direct gene expression data were not included, all generated strains, but the negative control, showed plasmid-based gene expression of the introduced genes, *adhE2*, and, where applicable, *bdhB*, from *C. acetobutylicum*, which was evidenced by a clear phenotypic change. Consequently, these modified *C. kluyveri* strains were capable of producing *n*-butanol and *n*-hexanol. In line with our expectations, both replacing P*_adhE2_* with the reportedly stronger P*_thl_* promoter and introducing a second, NADPH-dependent butanol dehydrogenase resulted in enhanced *n*-butanol and *n*-hexanol production. In two out of three growth experiments, we observed mutations in the promoter region of *C. kluyveri* pPthl_adhE2, resulting in the complete loss of *n*-butanol and *n*-hexanol production (Fig. S8 and S9B). In contrast, in the experiment without promoter mutations, this strain produced alcohols but exhibited an extended lag phase when the reportedly strong P*_thl_* promoter, which controlled the expression of only *adhE2*, was utilized. We did not detect these effects when the expression of *adhE2* was controlled by its native promoter P*_adhE2_*, indicating that the combination of high expression and this enzyme caused cellular stress and created selective pressure to inhibit its expression. We hypothesize that this stress may result from intracellular accumulation of toxic aldehydes. AdhE2 catalyzes both the reduction of acyl-CoA to aldehyde and, subsequently, to alcohol. If the enzyme has a higher affinity toward the first reaction and it is not localized within the native bacterial microcompartments in *C. kluyveri*, protecting the cells from aldehyde toxicity (53), the aldehydes may accumulate in the cytoplasm. The potential accumulation could explain the selection for mutations. Supporting this interpretation, coexpression of BdhB, which exclusively catalyzes the conversion of aldehydes to alcohols, prevented mutations and stabilized the growth phenotype. We propose that BdhB mitigates the aldehyde toxicity by promoting its faster conversion to less harmful alcohols, even under high expression of *adhE2*.

The batch experiment provided compelling evidence supporting the hypothesis that the combination of the P*_thl_* promoter and solely the *adhE2* gene exhibits toxicity. This toxicity was demonstrated conclusively by metabolic impairment: across the two other independent growth experiments, none of the *C. kluyveri* pPthl_adhE2 replicates produced detectable levels of either *n*-butanol or *n*-hexanol. In contrast, alcohol production was observed in only one experiment. Notably, the deletion identified in the P*_thl_* region (Fig. S9A) in this context suggests a selection pressure against the expression of *adhE2*. Sequencing of the alcohol-producing *C. kluyveri* pPthl_adhE2 (Fig. S9B) revealed mixed sequencing signals in the promoter region, indicating partial mutation. Because most of the cells were still unmutated, we attribute alcohol production to this majority. Given these findings, we cannot rule out that an unmutated pure culture of *C. kluyveri* pPthl_adhE2 could have performed better. Nonetheless, while using a stronger promoter enhanced the production of *n*-hexanol, adding the butanol dehydrogenase presumably helped accelerate aldehyde removal. This prevented toxic accumulation and resulted in a stable, mutation-free strain.

Furthermore, *C. kluyveri* pPthl_adhE2_bdhB produced less *n*-caproate despite this strain reaching the highest OD_600_ of all strains. This result may reflect a more balanced use of the cofactors NADH and NADPH. In this strain, alcohol production is catalyzed by two heterologous enzymes with distinct cofactor specificities, AdhE2 using NADH and BdhB NADPH. Distributing the reductive burden across both cofactors, the system prevents depletion of NADH alone, and thereby maintaining a more favorable NADH/NAD^+^ ratio. This balanced cofactor usage supports sustained alcohol production while avoiding redox imbalances (54). However, because NADH is also required for *n-*caproate formation (31), its partial diversion toward alcohol production may still reduce the flux toward carboxylate formation. The more balanced cofactor utilization of both NADH and NADPH could therefore contribute to higher alcohol production at the expense of *n*-caproate yield. These observations show the importance of redox cofactor allocation in engineered strains. Future studies involving mass balancing and cofactor dependent metabolic modeling would be valuable to further dissect and optimize these trade-offs. Notably, for redirecting bigger product fluxes in future studies, limitations might occur due to the changed redox balance.

The genetic system demonstrated in this study enabled plasmid-based gene expression, leading to heterologous production of *n*-butanol and *n*-hexanol by *C. kluyveri*. Besides expanding the product portfolio of this microbe, our research represents the first step toward developing a broader toolkit for *C. kluyveri*, including genome editing techniques (e.g., homologous recombination and CRISPR-based methods). Consequently, the genetic accessibility of the model microbe for chain elongation is a precondition for further research on the reverse β-oxidation pathway, which holds strong industrial potential. Therefore, gaining a deeper understanding of this pathway, both on a genetic level and in terms of the enzymes involved in their central energy metabolism, is crucial to enhancing its productivity.

## MATERIALS AND METHODS

### Microbial strains, media, and cultivation

*C. kluyveri* DSM555 was obtained from the DSMZ (Braunschweig, Germany) and used as the wild-type strain. Unless stated otherwise, all *C. kluyveri* strains in this study were cultivated in DSMZ52 medium (55) for which 2.5 g/L NaHCO_3_ was used to buffer the medium instead of Na_2_CO_3_. Cultures were grown in 100 mL medium with a headspace of 140 mL under an N_2_/CO_2_ (80/20) atmosphere. Both liquid cultures and plates (25 mL, stored at N_2_/CO_2_ [80/20] atmosphere in anoxic jars, as described by Fink et al. [56] containing 0.8% [vol/vol] Bacto agar) were incubated at 37°C until visible growth was observed. When required, the medium was supplemented with 5 µg/mL thiamphenicol to select for the cells carrying the desired plasmid and with 10 µg/mL trimethoprim to suppress *E. coli*. All genetic manipulations of *C. kluyveri* were conducted under strict anoxic conditions.

Unless otherwise noted, *E. coli* HB101 pRK2013 and *E. coli* TOP10 strains were grown in liquid lysogeny broth (LB) medium (57) or on LB plates containing 1.5% agar. For plasmid selection and maintenance, the medium was supplemented with 100 µg/mL ampicillin (for liquid cultures), 100 µg/mL carbenicillin (for plates), 30 µg/mL chloramphenicol, or 30 µg/mL kanamycin. IPTG was added at a concentration of 500 µM to induce the expression of the methyltransferase encoded on pMeth.

### Optimization of the plating efficiency

The variables (Table S1) were systematically adjusted one at a time to determine the optimal plating conditions, while all other conditions remained identical. Each agar plate contained 25 mL of solidified medium. The resulting outcomes were compared to the previously identified best conditions. Both the modified condition and the previously best-performing combination of conditions were tested using the same culture conditions and inoculation volume (4% [vol/vol]) with simultaneous incubation. The inoculum was serially diluted by increasing the dilution factor by a magnitude of 10, reaching a final dilution of 1:100,000, and each dilution was plated in duplicate for both conditions. Colony counts were performed by capturing images using the UVP GelStudio system (Analytic Jena, Jena, Germany) and analyzed with the integrated colony-count software. The plating conditions yielding the highest colony count were determined to be the updated optimal combination, and the following variable was tested accordingly.

### Restriction digest

Ten milliliters of *C. kluyveri* culture (OD_600_: 0.6–0.8) was harvested by centrifugation (17,900 rcf for 3 min), washed twice with 5 mL phosphate-buffered saline (PBS) buffer (137 mM sodium chloride, 2.7 mM potassium chloride, 10 mM sodium phosphate dibasic, and 1.8 mM potassium phosphate monobasic), and resuspended in 300 µL PBS buffer. Cells were lysed using bead-beating (30 s, 6.5 m/s; FastPrep-24; MP BioMedicals, Santa Ana, CA, USA). The lysing procedure was performed four times, and in between, the cells were kept on ice for 3 min. The lysis was centrifuged at 2,000 × *g* for 20 min at 4°C. To assess the sensitivity of the respective plasmid to the native restriction enzymes of *C. kluyveri*, 5 µL of the supernatant was added to 13 µL plasmid (200 ng) and 2 µL rCutSmart buffer (New England Biolabs [NEB], Ipswich, MA, USA). The prepared reaction mix was stored at 37°C for 1–24 h. Results were analyzed via gel electrophoresis, and potential corresponding restriction sites were analyzed *in silico* with SnapGene (GSL Biotech LLC, San Diego, CA, USA).

### PacBio sequencing

High molecular weight DNA (25 ng/µL) was isolated with the NucleoSpin Microbial DNA Mini kit for DNA (Macherey-Nagel, Düren, Germany) and sheared using 35 kb settings with a Megaruptor 2 instrument (Diagenode, SA, Lüttich, Belgium). Average insert sizes were approximately 10 kb. Sheared fragments were used to prepare libraries with the HiFi SMRTbell Express Template Prep Kit 2.0 (Pacific Biosciences, Menlo Park, CA, USA). The libraries were size-selected with a BluePippin system (SageScience, Beverly, USA) with 17 kb cutoff in a 0.75% DF Marker S1 High-Pass 6–10 kb vs3 gel cassette (Biozym, Hamburg, Germany). The library was sequenced with Sequencing Primer v2 (Pacific Biosciences, #101-847-900) and 4 h of pre-extension time on a single SMRT Cell with the Sequel II system using Binding Kit 2.0.

### Methylation profiling

Reads were demultiplexed using Lima (58) and converted into high-fidelity (HiFi) reads retaining kinetic information with Circular Consensus Sequencing (59). BAM files were converted to FASTQ with bam2fastx (60). Reads shorter than 1,000 bp and the lowest 5% of reads based on quality scores were discarded using Filtlong (61). Trycycler (62) was used to subsample reads and generate a consensus sequence from 12 assemblies independently generated with Flye (63), Miniasm (64), and Raven (65). HiFi reads were then aligned with the consensus assembly using pbmm2 (66), and ipdSummary (67) was used to detect and classify DNA base modifications from the kinetic signatures. MotifMaker (68) was used to identify statistically significant methylated motifs, with a minimum score threshold of 60. Motifs were retained if they were identified for 90% or more possible sites in the genome. The native methyltransferase of *C. kluyveri*, responsible for methylating the critical CCGG sites, was identified using the REBASE database (42).

### Plasmid construction

Plasmids (Table S16) were constructed with *E. coli* TOP10 and the respective primers (Table S17). The pUC19 vector (69) served as the backbone for the methylation plasmid pMeth. The gene encoding the critical methyltransferase (CKL_2671) from *C. kluyveri* was PCR amplified (Q5 polymerase, NEB) from genomic DNA (NucleoSpin, Microbial DNA; Macherey-Nagel) and inserted into the multiple cloning site via Gibson Assembly (NEBuilder, NEB). The lambda t1 terminator was introduced downstream of the CKL_2671 gene via PCR, amplifying the plasmid with overlapping primers and transferring the linear DNA inside *E. coli*. The same method was applied to remove the nucleotide sequence between the promoter and the start codon of CKL_2671 to utilize the promoter present in the backbone (P*_lac_*). In the final step, the origin of replication (ORI) was exchanged from the pSC101 ORI to the p15a ORI (70) by Gibson Assembly (NEBuilder, NEB) to ensure a low copy number and compatibility with the shuttle vector plasmids for *C. kluyveri*. For *n*-butanol and *n*-hexanol production, pMTL83151 (40) served as the backbone for all shuttle vector plasmids. The DNA fragments *adhE2*, *bdhB*, P*_adhE2_*, and P*_thl_* from *C. acetobutylicum* were PCR amplified (Q5 polymerase, NEB) from genomic DNA, and the respective fragments were inserted into the plasmids via Gibson Assembly (NEBuilder, NEB).

### Triparental conjugational DNA transfer

We inoculated LB medium supplemented with ampicillin, chloramphenicol, and IPTG (to induce the expression of the methyltransferase) with the donor strain *E. coli* TOP10 carrying the desired plasmid and incubated the culture overnight. When both the donor strain and the helper strain *E. coli* HB101 pRK2013, which was grown in LB medium supplemented with 30 µL kanamycin, reached an OD_600_ of 0.6–0.8, 1 mL of each culture was harvested by centrifugation (17,900 *× g*, 3 min). The resulting pellets were suspended and mixed in PBS buffer. We again centrifuged the *E. coli* mixture (17,900 *× g*, 3 min) and transferred the received cell pellet into the anoxic chamber. Subsequently, 2 mL of mid-exponential *C. kluyveri* wild-type culture (OD_600_: 0.4–0.6) was harvested via centrifugation (9,800 *× g*, 4 min) and resuspended in 50 µL RCM (36) under an N_2_ atmosphere. This cell suspension was used to resuspend the *E. coli* cell pellet. The cell mixture was then spot-plated on RCM plates (1.5% [vol/vol] agar) containing 500 µM IPTG and 257 mM ethanol (1.5% [vol/vol]). Plates were incubated at 37°C in an N_2_/CO_2_ (80/20) atmosphere for up to 120 h. After mating, cells from the mating spots were transferred via streaking onto DSMZ52 plates containing thiamphenicol to select for the respective plasmid. After cells had grown on the first selective plate, they were streaked onto a thiamphenicol and trimethoprim-containing DSMZ52 plate to select against *E. coli* strains. The resulting colonies were then further analyzed.

### Molecular methods for analysis of genetically modified *C. kluyveri*

Direct colony PCR was conducted on both colonies and liquid cultures to confirm the presence of the respective plasmid in *C. kluyveri* cells. Individual colonies were picked and resuspended in 25 µL of 10 mM NaOH to extract the DNA template. For PCR performed on liquid cultures, 100 µL of culture was sampled. In both cases, the cells were lysed at 98°C for 10 min using a ThermoMixer C (Eppendorf, Hamburg, Germany). For the PCR reactions, 1 µL of the lysate was utilized in two separate 10 µL PCR reactions (PhirePlant; Thermo Scientific, Waltham, USA): one using plasmid-specific primers and the other using *C. kluyveri*-specific primers. The results of the reactions were analyzed via gel electrophoresis. Additionally, a 30 µL PCR reaction targeting the 16S rRNA gene was performed using 3 µL of the lysate as a template. The resulting PCR product was purified using the QIAquick PCR Purification Kit (QIAGEN, Venlo, Netherlands) and subsequently analyzed via Sanger sequencing (GENEWIZ, Leipzig, Germany) to verify a pure culture of *C. kluyveri*.

### Analysis of compound concentration

To determine the concentrations of carboxylates (e.g., acetate, *n*-butyrate, and *n*-caproate) and alcohols (*n*-butanol and *n*-hexanol), an Agilent 7890B gas chromatograph (Agilent Technologies, Inc., Santa Clara, CA, USA) equipped with a DB-Fatwax UI capillary column (30 m × 0.25 mm × 0.25 µm) and flame ionization detector was used. Hydrogen served as the carrier gas, and the temperature program consisted of an initial temperature of 80°C for 30 s, ramped at 20°C/min to 180°C, followed by a 5-min hold. Injection and detector temperatures were set at 200°C and 250°C, respectively. Ethyl lactate was used as an internal standard at a concentration of 2 mM for quantifying carboxylates. The sampling volume was 1 mL of culture that was centrifuged (17,900 × *g*, 3 min) and from which we stored the supernatant at −20°C until further analysis. Before measuring, the samples were thawed and filtered through a 0.2 µm polyvinylidene difluoride sterile filter. For the analysis, samples were diluted at ratios of 1:2 for *n*-butanol and *n*-hexanol and 1:50 for MCC measurements.

For ethanol concentration measurements, undiluted samples were analyzed using the LC-20 high-performance liquid chromatography system (Shimadzu, Nishinokyo Kuwabara-cho, Japan). The system was equipped with an Aminex-HPX-87H column, and 5 mM sulfuric acid was utilized as the mobile phase at a flow rate of 0.6 mL/min (LC-20AD). The oven temperature was maintained at 65°C (CTO-20AC), and the samples were cooled at 15°C in the autosampler unit.

## SUPPLEMENTAL MATERIAL

Supplemental material aem.00058-26-s0001.pdf Supplemental methods, Fig. S1 to S9, and Tables S1 to S18.

ASM does not own the copyrights to Supplemental Material that may be linked to, or accessed through, an article. The authors have granted ASM a non-exclusive, world-wide license to publish the Supplemental Material files. Please contact the corresponding author directly for reuse.
