# Supplementary material for: Genetic modification of Clostridium kluyveri for heterologous n-butanol and n-hexanol production
Source: Appl Environ Microbiol. 2026 Mar 13;92(4):e00058-26. doi: 10.1128/aem.00058-26 (PMC13101501; doi:10.1128/aem.00058-26)
Supplement: Supplemental material — Supplemental methods, Fig. S1 to S9, and Tables S1 to S18. [file aem.00058-26-s0001.pdf]

SUPPORTING INFORMATION

Growth curve of wild-type *C. kluyveri*

To support the interpretation of growth phases referenced in the main text, we provide a representative growth curve of wild-type *C. kluyveri* (**Figure S1**). This curve shows the OD600 values that corresponding to early (0.2-0.4), mid (0.4-0.6), and late exponential phase (0.6-0.8).

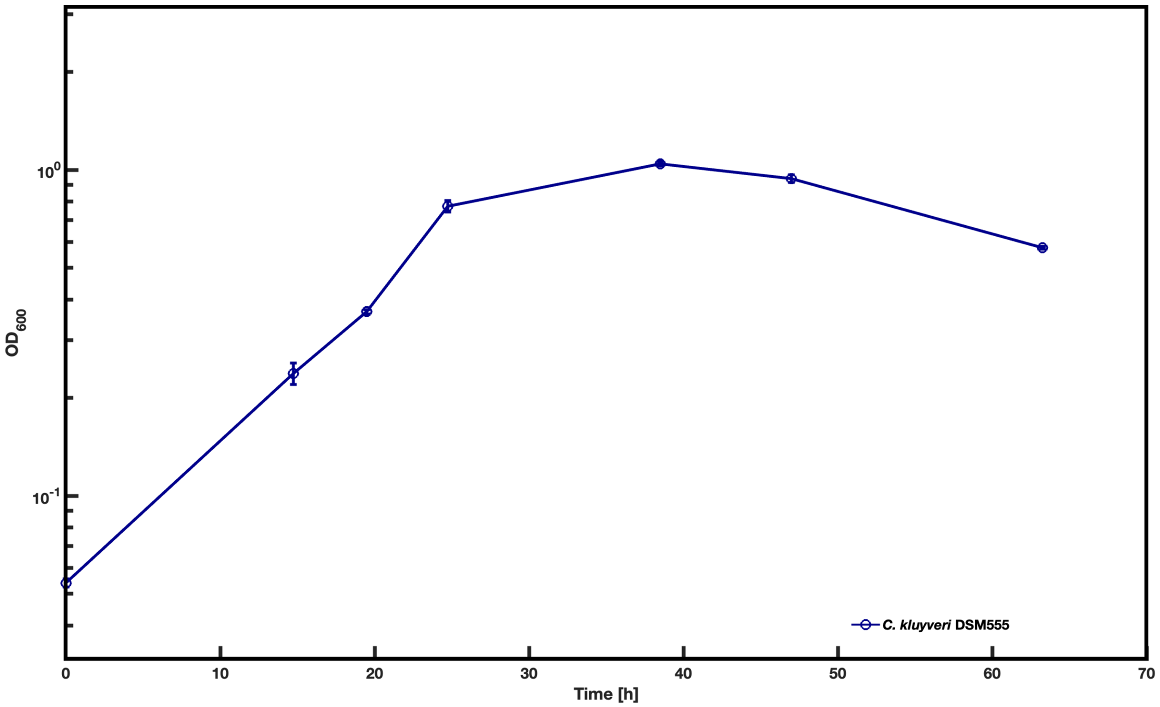

Figure S1. Growth curve of wild-type *C. kluyveri* in DSMZ52 medium.

Plating efficiency

The plating efficiency was optimized through a stepwise approach, where one condition was exchanged at a time while using same pre-culture for each comparison (**Table S1**). Due to experimental constraints, two biological replicates were performed per condition within the same anoxic jar to ensure strict parallel testing under identical conditions. Colony forming units (CFU) were counted for each replicate, and the mean value of these replicates is reported. The better-

performing condition of each comparison was designated as the current best condition, which then served as a control for the next round, where again a single factor was changed. If the new combination resulted in improved results, it was adopted as the updated best condition. Due to the limited number of replicates, statistical measures such as standard deviations were not calculated. This iterative process, conducted with control of variables, led to progressive improvements in plating efficiency over successive rounds.

We observed the biggest positive impact on the plating efficiency by supplementing a 10x concentration of the seven-vitamin solution, compared to the concentration used for cultivating *C. kluyveri* in liquid culture. We refer to this modified medium as DSMZ52*. The different conditions that we tested and their respective outcome are shown in **Table S1**. In order to enhance gas exchange during incubation, sterile metal paper clips were placed at the edge of the petri dish to lift the lid slightly (1). We expected this setup to improve CO_2 supply and removal of produced hydrogen.

Table S2 shows the plating efficiency of the standard conditions applied in this study.

Table S1. Optimization of the plating efficiency. Each line represents an independent and individual experiment in which two conditions were tested, respectively. Experiments are listed chronologically, meaning that the plating efficiency stays constant with every line or is improved. DSMZ52* means that DSMZ52 medium with 10x vitamin supplementation was used. From all conditions tested, pour plating a culture in its early exponential phase in DSMZ52* with 0.8% agar, 0.6 bar overpressure (N_2/CO_2), and a filter paper soaked with ethanol showed the best plating result.

Dilution factor	Current best combination	CFU per plate	Adapted condition	CFU per plate
1:100	Spread plating on DSMZ52	563	Spread plating on DSMZ52 with 10x vitamin supplementation	1,095
1:1,000	Spread plating on DSMZ52*	707	Spread plating on DSMZ52*, with paperclips for increased ventilation of CO2	148
1:100	Spread plating on DSMZ52*	1,001	Spread plating on RCM	402
1:1,000	Spread plating on DSMZ52*	980	Pour plating in DSMZ52*in 0.8% agar	1,056
1:100	Pour plating in DSMZ52* in 0.8% agar	944	Pour plating in DSMZ52* in 0.8% agar, additional EtOH in the headspace	141
1:10,000	Pour plating in DSMZ52* 0.8% agar, early exponential phase	678	Pour plating in DSMZ52 * in 0.8% agar, late exponential phase	216
1:10,000	Pour plating in DSMZ52* in 0.8% agar, early exponential phase	577	Pour plating in DSMZ52* in 0.8% agar, early exponential	748

			phase, at 0.6 bar overpressure	
1:10,000	Pour plating in DSMZ52* in 0.8% agar, early exponential phase, at 0.6 bar overpressure	551	Pour plating in DSMZ52* in 0.8% agar, early exponential phase, at 0.6 bar overpressure, with a filter paper soaked with EtOH inside the petri dish	579

*CFU: colony forming units per plate

DSMZ52: DSMZ52 medium with 10x seven-vitamin solution

Table S2. 50 µL of a 1:10,000 dilution from a mid-exponential culture (OD600: 0.3) were spread-plated on DSMZ52 medium. CFUs were counted for each replicate, and the corresponding plating efficiency was calculated. The average plating efficiency across replicates was 77% ± 15.14%.

	Number of CFUs	Plating efficiency
Replicate 1	148	91.08%
Replicate 2	113	69.53%
Replicate 3	97	59.69%
Replicate 4	144	88.62%

Transformation efficiency

Transformation efficiencies after 24 h, 48 h, and 72 h are summarized in **Table S3.** Although the highest number of colonies was observed after 48 h of mating, the transformation efficiency was highest after 24 h, normalized to account for cell growth on the mating plate. The transformation efficiencies were determined using biological triplicates.

The transformation efficiency for the recipient cells was calculated according to the following equation:

$$\frac{\boldsymbol{Counted\ CFUs}}{\boldsymbol{Plating\ efficiency} \times \boldsymbol{Cells\ plated} \times \boldsymbol{Fraction\ of\ recovered\ cells} \times \boldsymbol{Number\ of\ cell\ divisions}}$$

Where:

Counted CFUs: the number of colonies observed on selective medium after conjugation

Plating efficiency: the fraction of cells that form colonies when plated (determined in Table S2)

Cells plated: the number of cells applied on the mating plate

Fraction of recovered cells: the proportion of cells successfully recovered from the mating plate (we assumed a loss of 10%)

Number of cell divisions: the number of cell divisions that occurred during the mating time, utilized to normalize the efficiency per original recipient cell.

Table S3. Transformation efficiencies after different mating times, including the respective standard deviation (STDV).

Mating time	Transformation efficiency	STDV
24 h	2.36×10^{-7}	6.08×10^{-8}
48 h	1.97×10^{-7}	6.40×10^{-8}
72 h	1.08×10^{-7}	2.61×10^{-8}

Determination of the minimal inhibitory concentration of thiamphenicol for *C. kluyveri*

Wild-type cells of *C. kluyveri* were grown in liquid DSMZ52 medium at different thiamphenicol concentrations to determine the minimum inhibitory concentration (MIC). After one week, we observed growth up to a thiamphenicol concentration of 2 µg/mL, suggesting that 3 µg/mL was the MIC (**Figure S2**). However, to ensure no wild-type cells would grow, for example, due to spontaneous resistance, we decided to use a thiamphenicol concentration of 5 µg/mL to select the plasmid-carrying cells in liquid medium and 5 µg/mL on plates. The MIC determination was performed in biological duplicates and yielded identical results. Furthermore, our findings are consistent with thiamphenicol concentrations used for mutant selection in other published studies (2-4).

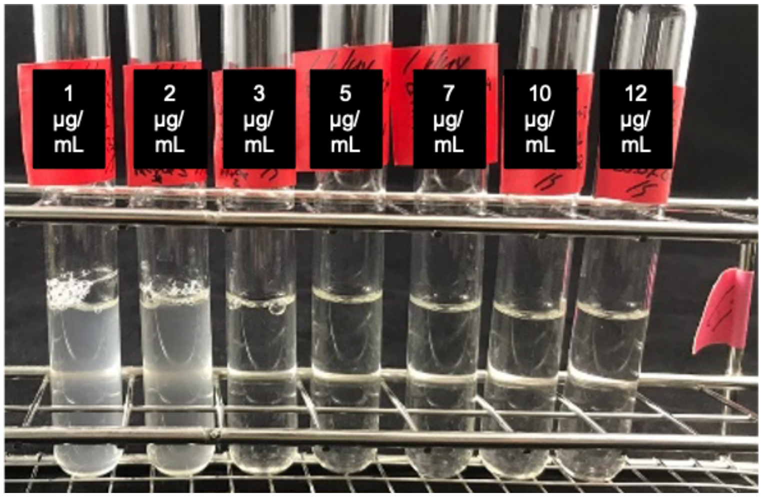

Figure S2. Determination of the MIC of thiamphenicol for *C. kluyveri*.

Native methylation pattern of *C. kluyve*ri DSM555

Sequencing of the methylation pattern of the genomic DNA of *C. kluyveri* DSM555 revealed methylation at specific sites (**Table S2)**. Analysis of the last three motif strings indicates the presence of N5-methylcytosin (m5C) modifications. Upon comparison, these motifs were determined to represent a common sequence and were thus grouped as CCGG. To ensure high-confidence results and eliminate false positives, we applied a stringent minimum quality threshold of 60 instead of the default value 40. This was feasible due to the high sequencing depth of the dataset. The associated statistical analysis was performed with the PacBio ipdSummary software, which uses a one-sample t-test (**Table S2)**.

Table S4. Results of the methylome analysis of *C. kluyveri* DSM555.

Motif String	Center Position	Modification Type	Fraction	N Detected	N Genome	Mean Score	Mean IpdRatio	Mean Coverage	Objective Score
GATC	1	m6A	1	10802	10802	761.7543	4.966895	622.5523	8228470
GWTAAT	4	m6A	0.991331	8005	8075	514.6464	3.7449944	615.75604	4087588.5
CCAAG	3	m6A	0.999780	4540	4541	588.4729	4.296277	615.4355	2671137.2
CAAAAAR	5	m6A	0.994054	3678	3700	383.9848	3.34622	611.4127	1404736.1
WDTTCCGGB	5	m5C	0.360434	133	369	124.7594	2.137143	560.6917	6623.1973
HNNNNNNNNNAAAW CCGG	15	m5C	0.288889	52	180	108.8846	2.0321157	545.53845	1851.9431
CCGGTAC	1	m5C	0.2875	46	160	111.6957	2.127174	600.26086	1673.2782

Digestion of pMTL83151 with *C. klu*yveri lysate

Comparing the detected methylation pattern of *C. kluyveri* to the observed digestion of pMTL83151, the CCGG methylation corresponded best to the observed restriction pattern. Therefore, we predicted the fragmentation of pMTL83151 at all CCGG sites and the resulting fragments are shown in **Table S5**. Fragments 2-5 are observed as one band on the gel, showing a threefold increased signal. Fragments shorter than 468 bp could not be identified on the gel due to their reduced fragment size, resulting in lower fluorescence intensity.

Table S5. Predicted fragments of pMTL83151 digested at CCGG sites. Fragments are sorted according to their respective size and visibility on the gel.

Fragment number	Fragment size (bp)	Visibility on gel
1	1,899	Yes
2	485	Yes
3	483	Yes
4	468	Yes
5	293	No
6	241	No
7	190	No
8	147	No
9	126	No
10	108	No
11	26	No
12	10	No

Construction of the methylation plasmid pMeth

The gene of the critical methyltransferase (CKL_2671) was PCR amplified from *C. kluyveri* genomic DNA and cloned into the multiple cloning site of pUC19. The T7 terminator was inserted into the plasmid and the ORI of pUC19 was replaced with the p15A ORI to generate the final plasmid pMeth (**Figure S3**).

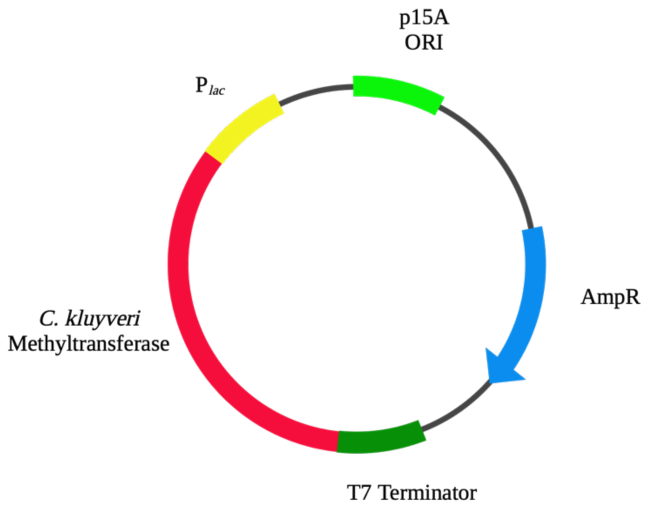

Figure S3. Plasmid map of pMeth based on pUC19.

Growth optimization of *E. coli* HB101 on RCM plates containing ethanol

In order to provide optimal conditions for both *E. coli* as donor and helper strain and *C. kluyveri* DSM555 to enable conjugation, we tested growth of *E. coli HB101* on RCM medium containing different ethanol concentrations (86-342 mM, 0.5-2.0% [v/v]) using biological duplicates. *E. coli* HB101 pRK2013 showed unrestricted growth up to a concentration of 257 mM of ethanol (Figure SXA) and restricted growth at 342 mM ethanol. Given that we cultivated *C. kluyveri* in 342 mM ethanol, we selected the highest ethanol concentration (257 mM) at which *E. coli* exhibited robust growth for the conjugation procedure.

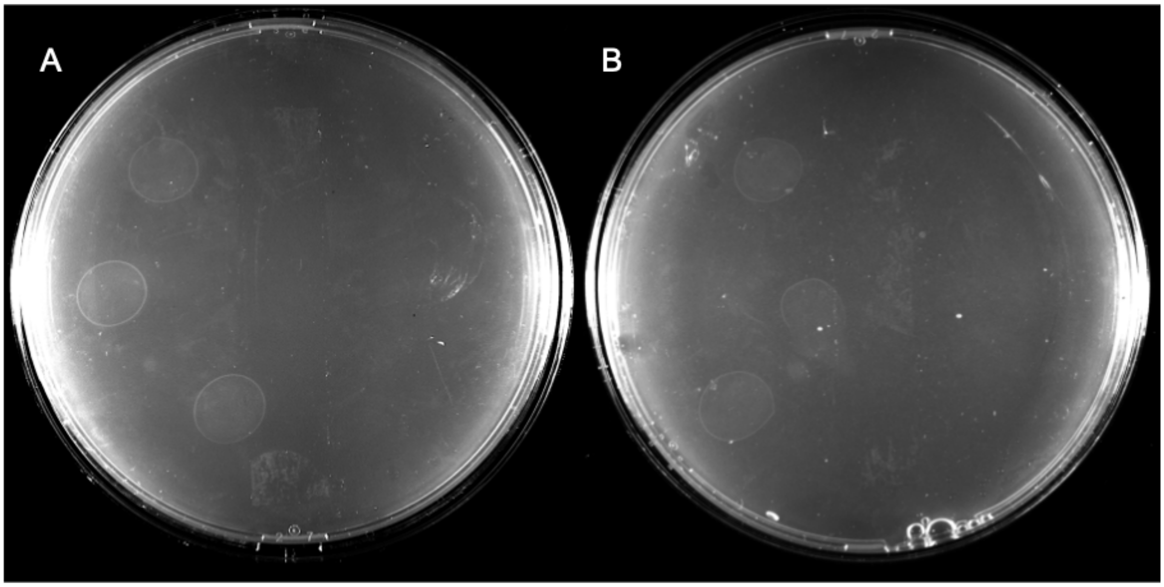

Figure S4. Growth of *E. coli* HB101 on RCM agar containing different ethanol concentrations. A) *E. coli* HB101 pRK2013 showed unrestricted growth up to 257 mM ethanol. B) At 342 mM ethanol, *E. coli* HB101 pRK2013 showed reduced growth.

Selection for plasmids

To identify the ideal mating time to obtain *C. kluyveri* mutants, we restreaked the potentially pMTL83151-carrying cells after 24, 48, 72, 96, and 120 h of mating. After 24 h, we observed individual colonies (**Fig. S5**).

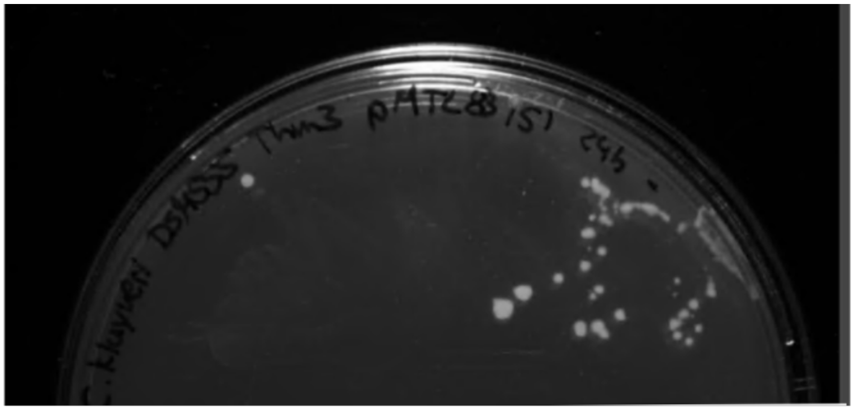

Figure S5. Growth of *C. kluyveri* colonies on selective plates after 24 h mating time.

Verification of pMTL83151 inside *C. kluyveri* cells

We screened 18 *C. kluyveri* colonies (lanes 1-18) for the presence of the plasmid pMTL83151 and verified its presence via colony PCR of the respective clones. Negative and positive (fragment size: ~1 kbp) controls are shown on the right side. The band corresponding to a longer fragment in the positive control results from the circular pMTL83151 plasmid, which served as template. B) Sanger Sequencing results of the 16S rRNA gene from the screened clones. All clones showed clear signals, confirming pure *C. kluyveri* pMTL83151 cultures. We show only one of the sequencing results here, which represents all tested clones.

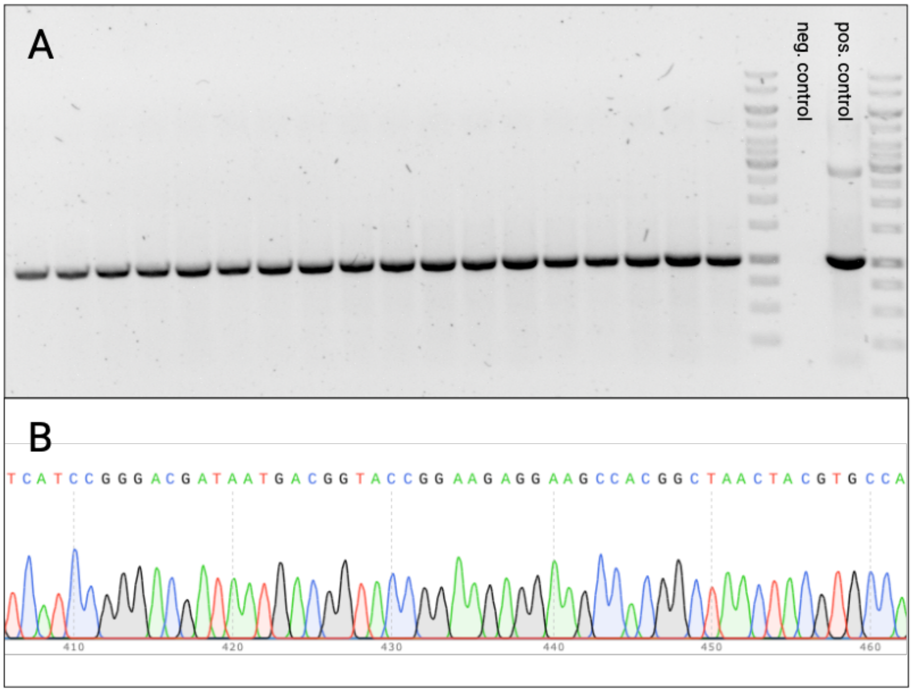

Figure S6. A) We screened 18 *C. kluy*veri colonies (lanes 1-18) for the presence of the plasmid pMTL83151 and verified its presence *via* colony PCR of the respective clones (expected fragment size: 1,001 bp, observed fragment size: ~1,000 bp). Negative (nuclease-free water) and positive (pMTL83151) controls are shown on the right side. The band corresponding to a longer fragment in the positive control results from the circular pMTL83151 plasmid, which served as template. B) Sanger Sequencing results of the 16S rRNA gene from the screened clones. All clones showed clear

signals, confirming pure *C. kluyveri* pMTL83151 cultures. We show only one of the sequencing results here, which represents all tested clones. Created with BioRender.com.

Growth rates and doubling times of the generated mutant strains

Growth rates and doubling times of the growth experiment, including their standard deviations, are provided in **Table S6**. Due to the considerable variation in lag phases between the biological replicates of *C. kluyveri* pPthl_adhE2, we separately calculated the values for each biological replicates. **Table S6** shows the resulting mean values and standard deviations.

Table S6. Growth rate μ and doubling times of the generated mutant strains including the respective standard deviation.

Strain	Growth rate μ	STDV μ	Doubling time	STDV Doubling time
C. kluyveri pMTL83151	0.111	0.009	6.296	0.566
C. kluyveri pPadhE2_adhE2	0.141	0.005	4.916	0.174
C. kluyveri pPthl_adhE2	0.088	0.015	8.023	1.372
C. kluyveri pPthl_adhE2_bdhB	0.151	0.006	4.594	0.190

Growth, substrate consumption, and product formation of the individual replicates of *C. kluyveri* pPthl_adhE2

The replicates of *C. kluyveri* pPthl_adhE2 showed a strong deviation in their respective lag phases, which caused lowered mean values of cell growth, substrate conversion, and product formation. **Figure S7** shows the data for each replicate of *C. kluyveri* pPthl_adhE2 individually.

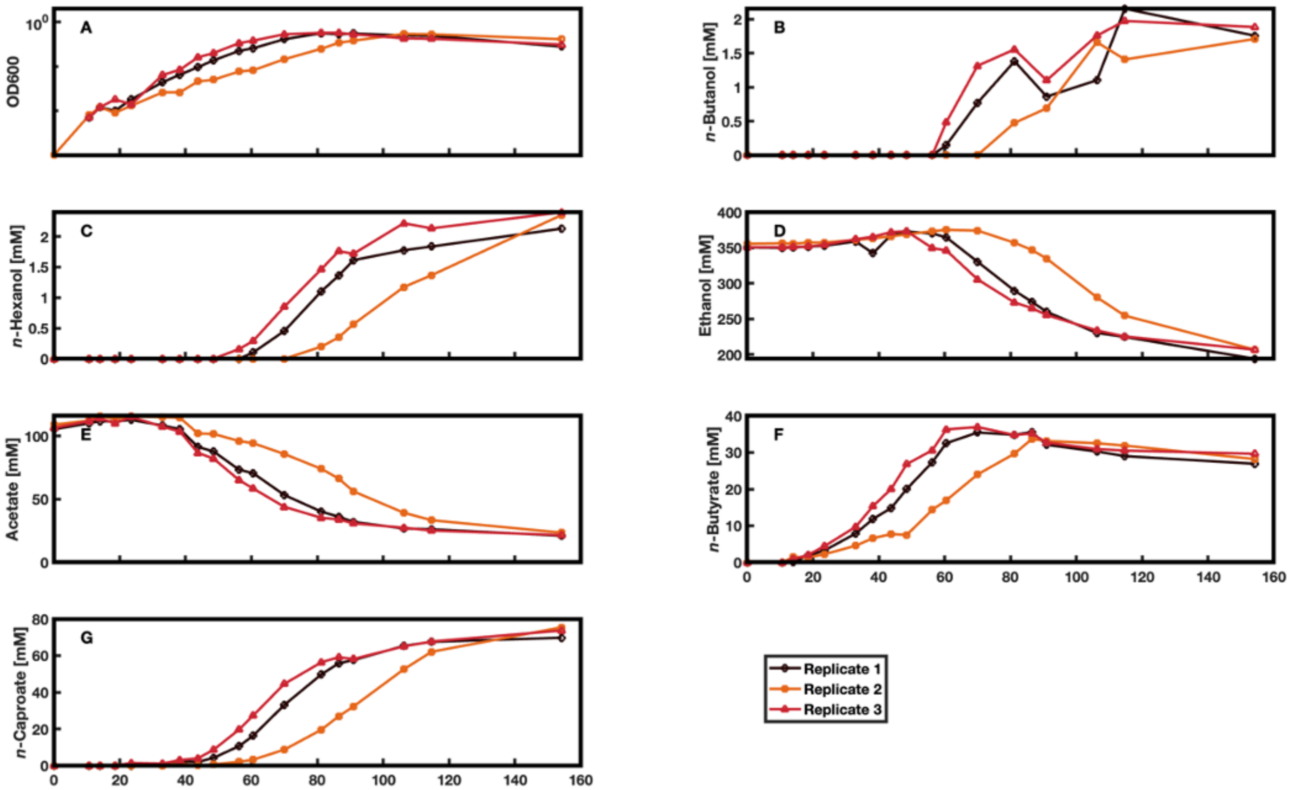

Figure S7. Growth behaviour, substrate consumption, and product formation of the biological replicates of *C. kluyveri* pPthl_adhE2 over time. A) depicts cell growth represented by the OD600. B) shows the *n*-butanol production. C) displays the *n*-hexanol formation. D) demontstrates the acetate consumption. E) presents the ethanol consumption. F) shows the *n*-butyrate formation. G) shows the *n*-caproate production.

Production of *n*-butanol and *n*-hexanol during growth experiments with mutated *C. kluyvrei* pPthl_adhE2

In two out of the three growth experiments, *C. kluyv*eri pPthl_adhE2 showed mutations in the promoter regions, resulting in inability to produce *n*-butanol and *n*-hexnol. However, *C. kluyver*i pPadhE2_adhE2 and *C. kluyveri* pPthl_adhE2_bdhB showed reliable growth throughout all three performed experiments (**Figure S8**).

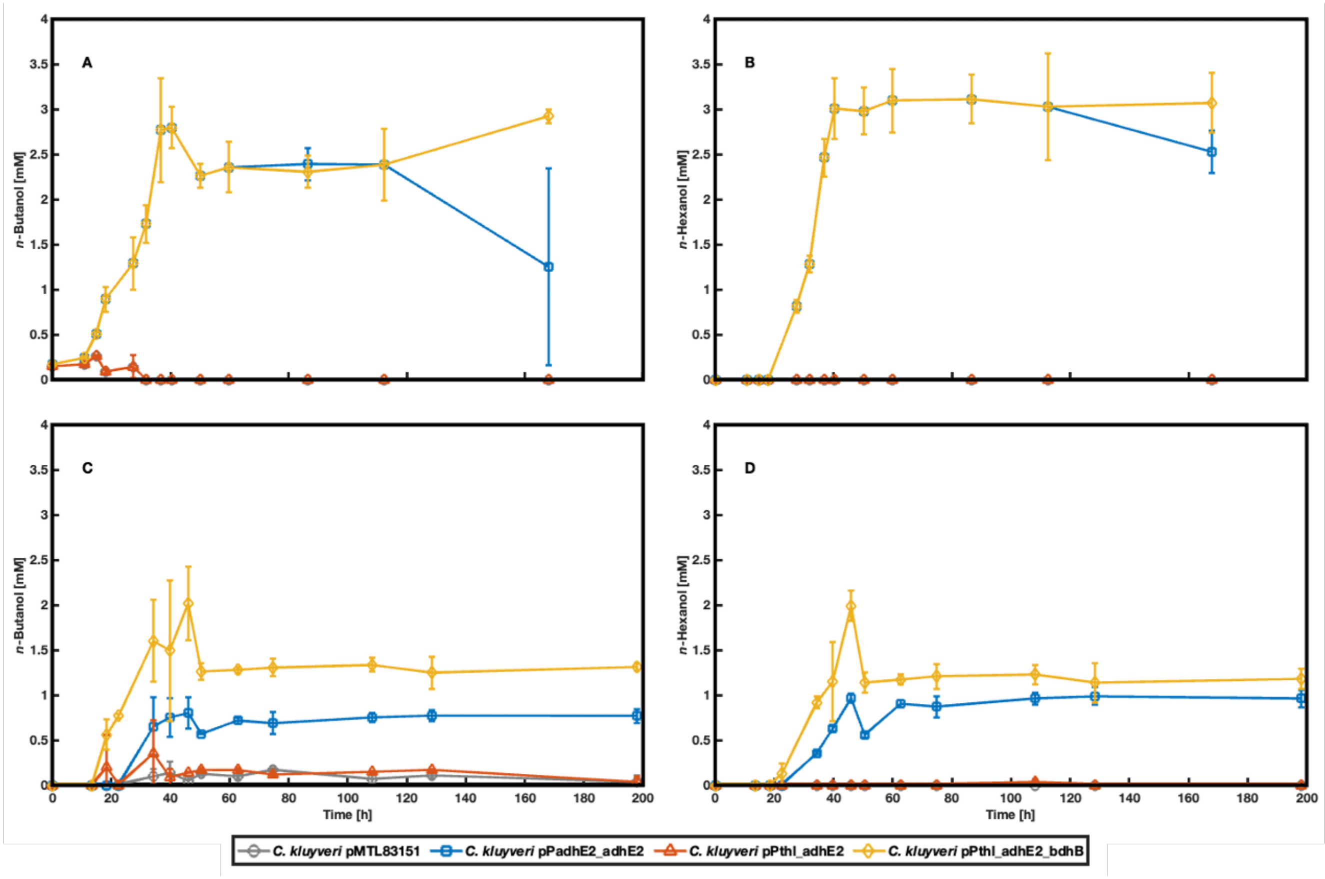

Figure S8. *n*-Butanol and *n*-hexanol production of the experiments showing a mutation in the promoter region of the plasmid pPthl_adhE2 leading to the inability to produce the alcohols. The experiment was performed in biological triplicates and the error bars represent standard deviations. A) and B) show the *n*-butanol and *n*-hexanol production of the first growth experiment and C) and D) of the second growth experiment where we observed this outcome.

Sanger Sequencing of P_{thl} in *C. kluyveri* pPthl_adhE2 cells

Sanger Sequencing of *C. kluyveri* pPthl_adhE2 revealed mutations in the promoter region P_{thl} of the *C. kluyveri* pPthl_adhE2, which did not produce any longer-chain alcohols than ethanol (**Figure S9A**). Also, the replicates of *C. kluyveri* pPthl_adhE2 seemed to have a fraction of mutated cells (marked with the red box) at the end of the experiment (**Figure S9B**).

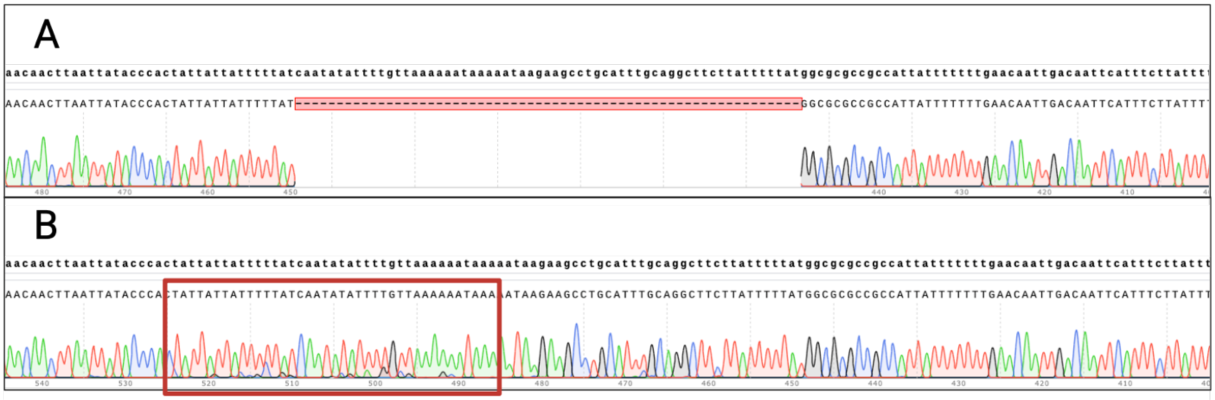

Figure S9. Sequencing results of the promoter region of *C. kluyveri* pPthl_adhE2. A) Deletion in the promoter region (P_{thl}) of adhE2 in *C. kluyveri* pPthl_adhE2 cultures, which did not produce *n*-butanol and *n*-hexanol. B) Shows the same, not deleted, promoter region of *C. kluyveri* pPthl_adhE2 cultures, which produced *n*-butanol and *n*-hexanol, at the end of the experiment. However, sequencing these cultures revealed mixed signals (especially inside the region marked with the red box), indicating that a fraction of the cells had mutated during the growth experiment. Created with BioRender.com.

Maximum *n*-butanol concentration

Table S7 shows the maximum *n*-butanol concentration for each strain. Due to the considerable variation in lag phases between the biological replicates of *C. kluyveri* pPthl_adhE2, the time points at which the maximum concentrations occurred varied between the replicates. To ensure consistency and comparability across all strains, we show the highest *n*-butanol concentrations measured for each replicate, regardless of the time at which it occurred.

Table S7. Maximum *n*-butanol concentration of the generated strains including the respective standard deviation (STDV).

	c_{max} *n*-Butanol [mM]				
	Replicate 1	Replicate 2	Replicate 3	Mean	STDV
C. kluyveri pMTL83151	0	0	0	0	0
C. kluyveri pPadhE2_adhE2	2.46	2.36	1.8	2.21	0.29
C. kluyveri pPthl_adhE2	2.16	1.66	2.39	2.07	0.30
C. kluyveri pPthl_adhE2_bdhB	4.24	3.64	4.23	4.04	0.28

Maximum *n*-hexanol concentration

Table S8 shows the maximum *n*-hexanol concentration for each strain. Due to the considerable variation in lag phases between the biological replicates of *C. kluyveri* pPthl_adhE2, the time points at which the maximum concentrations occurred varied between the replicates. To ensure consistency and comparability across all strains, we show the highest *n*-hexanol concentrations measured for each replicate, regardless of the time at which it occurred.

Table S8. Maximum *n*-hexanol concentration of the generated strains including the respective standard deviation (STDV).

	c_{max} *n*-Hexanol [mM]				
	Replicate 1	Replicate 2	Replicate 3	Mean	STDV
C. kluyveri pMTL83151	0	0	0	0	0
C. kluyveri pPadhE2_adhE2	2.38	2.36	1.75	2.16	0.29
C. kluyveri pPThl_adhE2	2.13	2.35	2.4	2.29	0.12
C. kluyveri pPthl_adhE2_bdhB	3.18	4.23	4.42	3.94	0.55

n-Butyrate concentration at initial alcohol synthesis

Table S9 presents the *n*-butyrate concentration corresponding to initial alcohol synthesis for each strain. Due to the considerable variation in lag phases between the biological replicates of *C. kluyveri* pPthl_adhE2, the time points at which initial alcohol production occurred varied between the replicates. To ensure consistency and comparability across all strains, we show the *n*-butyrate concentrations at initial alcohol formation measured for each replicate, regardless of the time at which it occurred.

Table S9. *n*-Butyrate concentration at initial alcohol synthesis of the generated strains including the respective standard deviation (STDV).

	$C_{n\text{-Butanol}}$ at initial alcohol synthesis [mM]				
	Replicate 1	Replicate 2	Replicate 3	Mean	STDV
C. kluyveri pMTL83151	-	-	-	-	-
C. kluyveri pPadhE2_adhE2	40.93	41.18	38.53	40.21	1.19
C. kluyveri pPThl_adhE2	32.54	29.69	36.31	32.85	2.71
C. kluyveri pPthl_adhE2_bdhB	32.67	34.59	35.48	34.25	1.17

n-Caproate concentration at initial alcohol synthesis

Table S10 presents the *n*-caproate concentration corresponding to initial alcohol synthesis for each strain. Due to the considerable variation in lag phases between the biological replicates of *C. kluyveri* pPthl_adhE2, the time points at which initial alcohol production occurred varied between the replicates. To ensure consistency and comparability across all strains, we show the *n*-caproate concentrations at initial alcohol formation measured for each replicate, regardless of the time at which it occurred.

Table S10. *n*-Caproate concentration at initial alcohol synthesis of the generated strains including the respective standard deviation (STDV).

	$C_{n\text{-Hexanol}}$ at initial alcohol synthesis [mM]				
	Replicate 1	Replicate 2	Replicate 3	Mean	STDV
C. kluyveri pMTL83151	-	-	-	-	-
C. kluyveri pPadhE2_adhE2	35.17	36.02	56.7	42.63	9.96
C. kluyveri pPThl_adhE2	16.19	19.54	27.29	21.01	4.65
C. kluyveri pPthl_adhE2_bdhB	19.36	21.97	23.87	21.73	1.85

Maximum *n*-butyrate concentration

Table S11 shows the maximum *n*-butyrate concentration for each strain. Due to the considerable variation in lag phases between the biological replicates of *C. kluyveri* pPthl_adhE2, the time points at which the maximum concentrations occurred varied between the replicates. To ensure consistency and comparability across all strains, we show the highest *n*-butyrate concentrations measured for each replicate, regardless of the time at which it occurred.

Table S11. Maximum *n*-butyrate concentration of the generated strains including the respective standard deviation (STDV).

	C_{max} *n*-Butyrate [mM]				
	Replicate 1	Replicate 2	Replicate 3	Mean	STDV
C. kluyveri pMTL83151	37.29	37.82	37.09	37.40	0.31
C. kluyveri pPadhE2_adhE2	40.93	41.18	45.55	42.55	2.12
C. kluyveri pPThl_adhE2	35.59	33.7	36.96	35.42	1.34
C. kluyveri pPthl_adhE2_bdhB	39.03	38.86	39.33	39.07	0.19

Maximum *n*-caproate concentration

Table S12 shows the maximum *n*-caproate concentration for each strain. Due to the considerable variation in lag phases between the biological replicates of *C. kluyveri* pPthl_adhE2, the time points at which the maximum concentrations occurred varied between the replicates. To ensure consistency and comparability across all strains, we show the highest *n*-caproate concentrations measured for each replicate, regardless of the time at which it occurred.

Table S12. Maximum *n*-caproate concentration of the generated strains including the respective standard deviation (STDV).

	C_{max} *n*-Caproate [mM]				
	Replicate 1	Replicate 2	Replicate 3	Mean	STDV
C. kluyveri pMTL83151	82.66	81.07	80.33	81.35	0.97
C. kluyveri pPadhE2_adhE2	73.54	73.66	75.56	74.25	0.93
C. kluyveri pPThl_adhE2	69.77	75.45	73.71	72.98	2.38
C. kluyveri pPthl_adhE2_bdhB	54.7	57.58	57.52	56.60	1.34

Substrate-to-product carbon conversion

Table S13 presents the substrate-to-product carbon conversion, calculated from the final concentrations of acetate and ethanol, for each biological replicate.

Table S13. Substrate-to-product carbon conversion of the generated strains including the respective standard deviation (STDV).

	Substrate-to-product carbon conversion [%]				
	Replicate 1	Replicate 2	Replicate 3	Mean	STDV
C. kluyveri pMTL83151	69.30	65.68	67.27	67.42	1.48
C. kluyveri pPadhE2_adhE2	63.01	63.20	62.66	62.96	0.22
C. kluyveri pPThl_adhE2	59.90	63.12	63.71	62.24	1.68
C. kluyveri pPthl_adhE2_bdhB	51.04	51.98	54.94	52.65	1.66

Final ethanol-to-acetate ratio

Table S14 presents the final ethanol-to-acetate ratio, calculated from the final concentrations of acetate and ethanol, for each biological replicate.

Table S14. Final ethanol-to-acetate ratio of the generated strains including the respective standard deviation (STDV).

	Final ethanol-to-acetate ratio				
	Replicate 1	Replicate 2	Replicate 3	Mean	STDV
C. kluyveri pMTL83151	8.28	8.17	8.33	8.26	0.07
C. kluyveri pPadhE2_adhE2	8.51	8.56	8.64	8.57	0.05
C. kluyveri pPThl_adhE2	9.19	8.79	9.58	9.19	0.32
C. kluyveri pPthl_adhE2_bdhB	6.63	5.87	6.17	6.23	0.31

Carbon balance of the generated strains

The carbon balance (**Table S15**) shows a carbon recovery close to 100% for all mutant strains. The small gap can be explained primarily by carbon incorporation into biomass. Further, CO2 uptake for amino acid biosynthesis was not considered in these calculations, as it has not been measured directly.

Table S15. Carbon balance of the generated strains including the respective standard deviation (STDV).

Mutant strains	Carbon balance [%]	STDV [%]
C. kluyveri pMTL83151	90.35	1.87
C. kluyveri pPadhE2_adhE2	95.04	0.36
C. kluyveri pPthl_adhE2	99.17	2.82
C. kluyveri pPthl_adhE2_bdhB	97.323	1.52

Plasmid list

Table S16. List of plasmids used in this study.

Name	Information	Reference
pMTL83151	Shuttle vector for *Clostridia*	Heap et al. 2009
pMeth	Methylation plasmid, encoding a methyltransferase of *C. kluyveri* DSM555 (CKL_2671) for methylation of plasmid DNA matching the methylation pattern of *C. kluyveri* (CCGG site)	This study
pUC19	Standard cloning vector	Yanisch-Perron et al. 1985
pPadhE2_adhE2	Alcohol production *via* bifunctional aldehyde/alcohol dehydrogenase AdhE2 from *C. acetobutylicum* (CA_P0035) controlled by the native promoter P_{adhE2}	This study
pPthl_adhE2	Alcohol production *via* bifunctional aldehyde/alcohol dehydrogenase AdhE2 from *C. acetobutylicum* (CA_P0035) controlled by the promoter P_{thl}	This study
pPthl_adhE2_bdhB	Alcohol production *via* bifunctional aldehyde/alcohol dehydrogenase	This study

	AdhE2 from *C. acetobutylicum* (CA_P0035) and the butanol dehydrogenase BdhB from *C. acetobutylicum* (CA_C3298) controlled by the promoter P_{thl}	

Primer list

Table S17. List of oligonucleotides used in this study.

Name	Sequence	Function
8F	AGAGTGTGATCCTGGCTCAG	Sequencing of the 16S rRNA gene region
1492r	GGTTTTTTTACGACTT	
CS-089	CTAGTCGACATGCGTGGTGGAAATCG	Amplification of CKL_2671
CS-090	CATCTAGATTATAAAGCTATAGTTTTTATTTTTTCAATATCCTG	
CS-095	CATTAGCGCAAGGTGATTTTTGTCTTCTTGCGCTAATTTTTTTCTAGAGGATCCCCGGG	T1 terminator exchange
CS-096	CCTTGCGCTAATGCTCTGTTACAGTTATAAAGCTATAGTTTTTATTTTTTCAATATCCTG	
CS-141	GAAACAGCTATGCGTGGTGGAAATCGAC	Gibson Assembly for p15a ORI
CS-142	CACCACGCATAGCTGTTTCCTGTGTGAAATTG	
CS-143	CCCGTAGAAAAGATCAAAGGATCTTCAAGATGATCTTCTTGAGATCGTTTTG	
CS-144	CGCGTTGCTGGCGTTCGTTTTTCCATAGGCTCCG	

CS-145	CGGAGCCTATGGAAAAACGAACGC CAGCAACGCG	Removal of remaining *lacZ* gene
CS-146	CAAAACGATCTCAAGAAGATCATC TTGAAGATCCTTTGATCTTTTCTAC GGG	
CS-112	CAAAACCTTCACGCTTGCCTATATT G	Sequencing pMeth
CS-147	GAGTCAGGCAACTATGGATGAAC	
CS-148	GAGTCAGTGAGCGAGGAAG	
CS-159	TTTTTATTGCCAAATACTAGCATAA AAATAAGAAGCCTGCAT	Gibson Assembly for P_{adhE2}_*adhE2*_terminator insert into pMTL83151
CS-160	GCTTTTTACTCCTGCTTGACATGGA TACAGCGGCC	
CS-161	ACCGCGGCCGCTGTATCCATGTCAA GCAGGAGTAAAAAGCG	
CS-162	GCAGGCTTCTTATTTTTATGCTAGT ATTTGGCAATAAAAATAGTTATAAT CATTAATTATTG	
CS-240	TTTTGATTTGTAACTTTCATATGAA CTAACCTCCTAAATTTTGATACGGG G	Gibson Assembly for P_{thl} insert

CS-241	TGCAGGCTTCTTATTTTTATTTTTTA ACAAAATATATTGATAAAAATAAT AATAGTGGGTATAATTAAG	
CS-242	TCAATATATTTTGTTAAAAAATAAA AATAAGAAGCCTGCATTTGCAGG	
CS-243	AATTTAGGAGGTTAGTTCATATGAA AGTTACAAATCAAAAAGAACTAAA ACAAAAGCTAAATG	
CS-236	TTTATCTACAATTTTTTTATACTCCC CAAGATATTAATGCTTT	
CS-237	TAAAATCATTTTAAAAAATAGTGGT TGATTTCGAATATTCAATACC	
CS-238	GAATATTCGAAATCAACCACTATTT TTTAAAATGATTTTATATAGATATC CTTAAGTTCACTTATAAG	Gibson Assembly for *bdhB_terminator* insert
CS-239	GCATTAATATCTTGGGGAGTATAAA AAAATTGTAGATAAAACTATTTTAT AAAATTTATCTACAAT	
EB00003594	GTACTGCTTCCAATACACAAAGGC	
CS-162	GCAGGCTTCTTATTTTTATGCTAGT ATTTGGCAATAAAAATAGTTATAAT CATTAATTATTG	Sequencing P_{adhE2}_*adhE2*, P_{thl}_*adhE2*, and P_{thl}_*adhE2_bdhB*
CS-227	CTGGATAGATGAGCCATCAATAG	

CS-228	GTGTTGCTGAAAGAAGGGAAAATATG	
CS-229	GATTATACTGATGAATTAGCCTTAAGAGC	
CS-237	TAAAATCATTTTAAAAAATAGTGGTTGATTTCGAATATTCAATACC	
MRS-040	CAAGGCAAGACCGATCG	
EB00002844	GCGGCCGCTCTTTCCTGCGTTATCCCCTG	Verification of pMTL83151-based plasmids
EB00002845	ACATGTGGTCATAGCTGTTTCCTGATAAAAAAATTG	

Bacterial strains

Table S18. List of bacterial strains used in this study.

Name	Source
C. acetobutylicum ATCC824	ATCC
C. kluyveri DSM555	DSMZ
C. kluyveri pMTL83151	This study
C. kluyveri pPadhE2_adhE2	This study
C. kluyveri pPthl_adhE2	This study
C. kluyveri pPthl_adhE2_bdhB	This study
E. coli TOP10 pMeth	This study
E. coli TOP10 pMeth pMTL83151	This study
E. coli TOP10 pPadhE2_adhE2	This study
E. coli TOP10 pMeth pPadhE2_adhE2	This study
E. coli TOP10 pPthl_adhE2	This study
E. coli TOP10 pMeth pPthl_adhE2	This study
E. coli TOP10 pPthl_adhE2_bdhB	This study
E. coli TOP10 pMeth pPthl_adhE2_bdhB	This study
E. coli HB101 pKR2013	DSMZ

Chemicals and suppliers

Table S18. List of chemicals used in this study and their respective suppliers.

Chemical	Supplier
Ampicilin	Carl Roth, Karlsruhe, Germany
Beef extract	Carl Roth, Karlsruhe, Germany
Calcium pantothenate	Carl Roth, Karlsruhe, Germany
Carbencillin	Merck, Burlington, MA, USA
Chloramphenicol	Carl Roth, Karlsruhe, Germany
$CoCl_2$ x 6 H_2O	Carl Roth, Karlsruhe, Germany
$CuCl_2$ x 2 H_2O	Carl Roth, Karlsruhe, Germany
D-(+)-biotin	Carl Roth, Karlsruhe, Germany
Ethanol absolute	Carl Roth, Karlsruhe, Germany
FeCl x 4 H_2O	Carl Roth, Karlsruhe, Germany
H_3BO_3	Carl Roth, Karlsruhe, Germany
HCl	Carl Roth, Karlsruhe, Germany
IPTG	Merck, Burlington, MA, USA
K_2HPO_4	Carl Roth, Karlsruhe, Germany
KH_2PO_4	Carl Roth, Karlsruhe, Germany
Kanamycin	VWR International, Radnor, PA, USA
L-Cystein	Carl Roth, Karlsruhe, Germany
$MnCl_2$ x 4 H_2O	Carl Roth, Karlsruhe, Germany
Na resazurin	Carl Roth, Karlsruhe, Germany
Na_2MoO_4 x 2 H_2O	Carl Roth, Karlsruhe, Germany

Na_2S (60%)	Carl Roth, Karlsruhe, Germany
Na_2SeO_3 x 5 H_2O	Carl Roth, Karlsruhe, Germany
Na_2WO_4 x 2 H_2O	Carl Roth, Karlsruhe, Germany
NaCl	Carl Roth, Karlsruhe, Germany
$NaHCO_3$	Carl Roth, Karlsruhe, Germany
NaOH	Carl Roth, Karlsruhe, Germany
NH_4Cl	Carl Roth, Karlsruhe, Germany
NiCl2 x 6 H_2O	Carl Roth, Karlsruhe, Germany
Nictonic acid	Carl Roth, Karlsruhe, Germany
p-Mnimobenzoic acid	Carl Roth, Karlsruhe, Germany
Peptone	Carl Roth, Karlsruhe, Germany
Potassium acetate	Carl Roth, Karlsruhe, Germany
Pyridoxine hydrochloride	Carl Roth, Karlsruhe, Germany
Solube starch	Carl Roth, Karlsruhe, Germany
Thiamine-HCl x 2 H_2O	Carl Roth, Karlsruhe, Germany
Thiamphenicol	Merck, Burlington, MA, USA
Trimethoprim	VWR International, Radnor, PA, USA
Vitamin B12	Carl Roth, Karlsruhe, Germany
Yeast extract	Carl Roth, Karlsruhe, Germany
$ZnCl_2$	Carl Roth, Karlsruhe, Germany

Plasmid sequences

pMTL83151: https://www.novoprolabs.com/vector/Vhaytmna

pUC19: https://www.addgene.org/50005/

pMeth: The gene of the methyltransferase (corresponding to CKL_2671) is displayed in blue and the promoter P_{lac} in green.

aagatgatcttcttgagatcgttttggtctgcgcgtaatctcttgctctgaaaacgaaaaaaccgccttgcagggcggtttttcgaaggttctctg
agctaccaactctttgaaccgaggtaactggcttggaggagcgcagtcaccaaaacttgtcctttcagtttagccttaaccggcgcatgacttc
aagactaactcctctaaatcaattaccagtggctgctgccagtggtgcttttgcatgtctttccgggttggactcaagacgatagttaccggata
aggcgcagcggtcggactgaacggggggttcgtgcatacagtccagcttggagcgaactgcctacccggaactgagtgtcaggcgtgga
atgagacaaacgcggccataacagcggaatgacaccggtaaaccgaaaggcaggaacaggagagcgcacgagggagccgccaggg
ggaaacgcctggtatctttatagtcctgtcgggtttcgccaccactgatttgagcgtcagatttcgtgatgcttgtcaggggggcggagcctat
ggaaaaacgaacgccagcaacgcggcctttttacggttcctggccttttgctggccttttgctcacatgttctttcctgcgttatcccctgattctg
tggataaccgtattaccgcctttgagtgagctgataccgctcgccgcagccgaacgaccgagcgcagcgagtcagtgagcgaggaagcg
gaagagcgcccaatacgcaaaccgcctctccccgcgcgttggccgattcattaatgcagctggcacgacaggtttcccgactggaaagcg
ggcagtgagcgcaacgcaattaatgtgagttagctcactcattaggcaccccaggctttacactttatgcttccggctcgtatgttgtgtggaatt
gtgagcggataacaatttcacacaggaaacagctatgcgtggtggaaatcgactcggtgcaggaaggaaggttattcctgagtcagaaaaa
aagaaaagaaaaagcgtgtatattacagataaactttacactagaattatggatactgacatcgaaaattgcaataatttcagtcaaaaatgtatg
gcattgattgaattagcaatggaaaatctaaataagaacaatcaagagcatagtgtgaaaaggaacaatatattgatggtgagagaatccaaa
tctacatacaacaaaacgaataataattttgaaaaacaaaaccttggaataaaattgacatttattgatttattcgctggtattggaggaattagatt
aggatttgaagacaaatatacgaaatgtgtatttagttctgaatgggataaatatgcagctcaaacgtatgaagctaattatggtgagaagcctc
atggtgatattacaaaaatcaacgaaaatgatattccagatcacgatgttttattggctggatttccttgtcagccgtttagcaatataggcaagc

gtgaaggttttgctcatgaaacgcaaggaacattatttttcgatgttcttaggatcctaaagaaaaagcaacctaagatgtttttgttagaaaatgtaaaagggcttttaacaaatgataatgggaatacatttcgagtcattttagacaatcttaagagtctaggatattccgtttttatgaagttatggatgcacaaaattttgggcttccacaaagacgtgaacgtattgtaattgtgggatttcatcctgatttaggcattaatgatttttcatttcctaaaggtaatcctgacaataaggttccaattaatgctattttagagcataatcctacagggtattcaatttccaagcgtttgcaagaaagttacttatttaaaaaggatgatgggaagccacagattgttgattttgatagcactatacaagttaacactttagtagctagttatcacaaaatacagcgtcttacaggaacatttgtaaaagacggagaaacagggcttcgattattcagcgaattagaactgaaacgtttgatgggtttccctgatgattttaaagttcctgtgtcaagaacacaaatgtataggcagtttggaaattctgttgctgttcccatgattaaagctgttgcagaagcaatgaaagagcgacttttgttggctgaaatgcaggatattgaaaaaataaaaactatagctttataactgtaacagagcattagcgcaaggtgatttttgtcttcttgcgctaatttttTctagaggatccccgggtaccgagctcgaattcactggccgtcgttttacaacgtcgtgactgggaaaaccctggcgttacccaacttaatcgccttgcagcacatccccctttcgccagctggcgtaatagcgaagaggcccgcaccgatcgcccttcccaacagttgcgcagcctgaatggcgaatggcgcctgatgcggtattttctccttacgcatctgtgcggtatttcacaccgcatatggtgcactctcagtacaatctgctctgatgccgcatagttaagccagccccgacacccgccaacacccgctgacgcgccctgacgggcttgtctgctcccggcatccgcttacagacaagctgtgaccgtctccgggagctgcatgtgtcagaggttttcaccgtcatcaccgaaacgcgcgagacgaaagggcctcgtgatacgcctatttttataggttaatgtcatgataataatggtttcttagacgtcaggtggcacttttcggggaaatgtgcgcggaacccctatttgtttatttttctaaatacattcaaatatgtatccgctcatgagacaataaccctgataaatgcttcaataatattgaaaaaggaagagtatgagtattcaacatttccgtgtcgcccttattcccttttttgcggcattttgccttcctgtttttgctcacccagaaacgctggtgaaagtaaaagatgctgaagatcagttgggtgcacgagtgggttacatcgaactggatctcaacagcggtaagatccttgagagttttcgccccgaagaacgttttccaatgatgagcacttttaaagttctgctatgtggcgcggtattatcccgtattgacgccgggcaagagcaactcggtcgccgcatacactattctcagaatgacttggttgagtactcaccagtcacagaaaagcatcttacggatggcatgacagtaagagaattatgcagtgctgccataaccatgagtgataacactgcggccaacttacttctgacaacgatcggaggaccgaaggagctaaccgcttttttgcacaacatgggggatcatgtaactcgccttgatcgttgggaaccggagctgaatgaagccataccaaacgacgagcgtgacaccacgatgcctgtagcaatggcaacaacgttgcgcaaactattaactggcgaactacttactctagcttcccggcaacaattaatagactggatggaggcggataaagttgcaggaccacttctgcgctcggcccttccggctggctggtttattgctgataaatctggagccggtgagcgtgggtctcgcggtatcattgcagcactggggccagatggtaagccctcccgtatcgtagttatctacacgacggggagtcaggcaactatggatgaacgaaatagacagatcgctgagataggtgcctcactgattaagcattggtaactgtcagaccaagttt

actcatatatactttagattgatttaaaacttcatttttaatttaaaaggatctaggtgaagatcctttttgataatctcatgaccaaaatcccttaacgtgagttttcgttccactgagcgtcagaccccgtagaaaagatcaaaggatcttc

<u>pPadhE2_adhE2</u>: The coding sequence of *adhE2* is displayed in orange, and the upstream region of the *adhE2* gene on the genome of *C. acetobutylicum* containing its native promoter is displayed in green.

cctgcaggataaaaaaattgtagataaattttataaaatagttttatctacaatttttttatcaggaaacagctatgaccgcggccgctgtatccatgtcaagcaggagtaaaaagcgaaaagacctgtaaagaattgtggagtaaaggatacaaggtaattaatattaaagatggtacagattcgtacaagcaaccacttgaaagataaaaaacaagagtaaaatgtaaaatagtctatgtgcttcatgaagctaatataatgaagcaaagactattttacattctttattttttaaaatgattttatatagatatccttaagttcacttataagtggatacctaggattagctgttgtacattggtcatcaaaagcaagctctgacattttatctagcgtattataaaaatctttttttatttattccagcggcacttatattttgtggaatactcaaatctatctttaactttgaaatagcttctattaaggctgttaccttttcggtatcgctagtaccctttaaattcaaatactctgcaatttcagcatattttctcttagcattaggagatttatattgagggaatgctgtttgctttgttggacagtctgtagcgttatatttaataacttcttctattaatacagcacaagcaattccatgtggaacgtgatgcattgccccaagtttatgagccattgaatggcatacacctaagaaagcatttgcaaatgccatccccgcaatattagaggcatgtgccatttttttctcttgcttcaatgtcgttagtcccatttttataggctctaggcaaatatttaaatatcatttttattgctcttaaggctaattcatcagtataatccgtagccataaccgaaacatatgcttctatagcatgaactaatgcatctattccagttgctgctgttaattttctaggcatatttaacattaattcagtatctattattgccatgtttggggtcaattcataagaagttaaagggtatttcattcctgtttcatcattagttataactgcaaaaggtgttgcctctgaaccggtaccagcagttgtaggaatagctactgaaatcgcctttgtacctaatttagggaaattgcatattctctttcttatatccataaagtttatagctagattttcaatttctgcttctggatattcatataacaagtgcataacctttgctgcatccattggcgatccaccaccaatagagattatagtatcaggttcaaagttaagcatttctttagcaccttttttttactgaatcaatagttggatcagatttaatatctgtaaatatactgtatttaatatctatctcatctagtacctttgttattttattaacatatccaagtttaaaaagatctttatctgttactataaaggctcttttcttattcatatcttttaattcttttaatgcaaatctaagacatccatatttaaaatatatttttttgtggcactttaaaccaaagcatattttcccttctttcagcaacacttttaatatttaataaatgtttaggctctacattttgcgatacagagtttcctccccaagtgccgcatccaagagtaaatgatggtgctatcgcaaaattgtataaatctccgcttgctccctgtgaagaaggcatgtt

aataaatgtccttgaagttttcattgctaatccaaattctttaactttatccttattgttttgtgaatctatatataaagatgacgtgtgtccacttccacctaattctattagcctttgtgcctttttttagagcttcatcaaaatccttaactttatacattgcaagtactggtgatagtttttcatgtgagaacagctcgctttttcaacagattgtacttcgcctataagtatctttgtagtttgaggaacttcaattcctgccattttagcaattatataagcagattttccaactatgtcagcattaatagctccatttttaaacatagtttcttttattttagctatttcattttgattgagtatatatgatcctcgttttacaaattcctctttaactttttcgtatattgaattcataactaatattgattgttcagaagcgcatattactccattgtcataagtctttgataaaattatggagcttactgccatatctatatctgcactctcatctattattgctggtgtatttcctgctccaacaccaattgcaggttttccagatgaataggccgctttaaccattgaaggacctcctgttgctaatattatatcagcttcactcatcaaatcttgagaaagttctattgatggctcatctatccagcctattatatttttaggtgctcctgctttaacagctgcatctaaaattaattttgctgcagcaattgtagatttttttgcacgtggatgtggtgaaaagaatattgcgtttcttgtttttaaagaaattaatgatttgaaaattgctgtggaagttggattagtagtaggaactatggctgcaacaattccaattggttcagcaacctttgttatgcctaaagaatcgtcatggtctattatgccacaagtttttttcatttttatatttattgtatatatattctgctgcaaaatgatttttttataattttatcttctacaagacctattcctgtttcttctactgctaatttagctaagtttattctttctttagctgcggctatggcacattgtttaaaaattttatcaacttgctcttgagtataggttgcaaacttcttttgcgcttctctcaattcatttagcttttgttttagttctttttgatttgtaactttcatttatatacactcctttataaaatttattattagtttgatatttaacaaactattgcaaataaaacttattgtattatataacacactttgatgccttataaattatagagtgtggtttccttacattatttacttctaatgtgataaaccgttgatatgtacatatttaacgcttgtatttctatagtaaaattatgcagtcattttattttctataatattattatataacagtaattaaagattgtcaaacatttaacaataattaatgattataactatttttattgccaaatactagcataaaaataagaagcctgcatttgcaggcttcttatttttatggcgcgccgccattattttttgaacaattgacaattcatttcttattttttattaagtgatagtcaaaaggcataacagtgctgaatagaaagaaatttacagaaaagaaaattatagaatttagtatgattaattatactcatttatgaatgtttaattgaatacaaaaaaaaatacttgttatgtattcaattacgggttaaaatatagacaagttgaaaaatttaataaaaaaataagtcctcagctcttatatattaagctaccaacttagtatataagccaaaacttaaatgtgctaccaacacatcaagccgttagagaactctatctatagcaatatttcaaatgtaccgacatacaagagaaacattaactatatatattcaatttatgagattatcttaacagatataaatgtaaattgcaataagtaagatttagaagtttatagcctttgtgtattggaagcagtacgcaaaggctttttatttgataaaaattagaagtatatttatttttcataattaatttatgaaaatgaaagggggtgagcaaagtgacagaggaaagcagtatcttatcaaataacaaggtattagcaatatcattattgactttagcagtaaacattatgacttttatagtgcttgtagctaagtagtacgaaaggggagctttaaaaagctccttggaatacatagaattcataaattaatttatgaaaagaagggcgtatatgaaaacttgtaaaaattgcaaagagtttattaaagatactgaaatatgcaaaatacattcgttgatgattcatgataaaacagtagcaacctattgcagtaaatacaatgagtcaagatgtttacataaagggaa

agtccaatgtattaattgttcaaagatgaaccgatatggatggtgtgccataaaaatgagatgttttacagaggaagaacagaaaaaagaacgt
acatgcattaaatattatgcaaggagctttaaaaaagctcatgtaaagaagagtaaaaagaaaaaataatttatttattaatttaatattgagagtg
ccgacacagtatgcactaaaaaatatatctgtggtgtagtgagccgatacaaaaggatagtcactcgcattttcataatacatcttatgttatgatt
atgtgtcggtgggacttcacgacgaaaacccacaataaaaaaagagttcggggtagggttaagcatagttgaggcaactaaacaatcaagc
taggatatgcagtagcagaccgtaaggtcgttgtttaggtgtgttgtaatacatacgctattaagatgtaaaaatacggataccaatgaaggga
aaagtataatttttggatgtagtttgtttgttcatctatgggcaaactacgtccaaagccgtttccaaatctgctaaaaagtatatcctttctaaaatc
aaagtcaagtatgaaatcataaataaagtttaattttgaagttattatgatattatgtttttctattaaaataaattaagtatatagaatagtttaataata
gtatatacttaatgtgataagtgtctgacagtgtcacagaaaggatgattgttatggattataagcggccggccagtgggcaagttgaaaaattc
acaaaaatgtggtataatatctttgttcattagagcgataaacttgaatttgagagggaacttagatggtatttgaaaaaattgataaaaatagttg
gaacagaaaagagtattttgaccactactttgcaagtgtaccttgtacctacagcatgaccgttaaagtggatatcacacaaataaaggaaaag
ggaatgaaactatatcctgcaatgctttattatattgcaatgattgtaaaccgccattcagagtttaggacggcaatcaatcaagatggtgaattg
gggatatatgatgagatgataccaagctatacaatatttcacaatgatactgaaacattttccagcctttggactgagtgtaagtctgactttaaat
catttttagcagattatgaaagtgatacgcaacggtatggaaacaatcatagaatggaaggaaagccaaatgctccggaaaacatttttaatgt
atctatgataccgtggtcaaccttcgatggctttaatctgaatttgcagaaaggatatgattatttgattcctatttttactatggggaaatattataaa
gaagataacaaaattatacttcctttggcaattcaagttcatcacgcagtatgtgacggatttcacatttgccgttttgtaaacgaattgcaggaat
tgataaatagttaacttcaggtttgtctgtaactaaaaacaagtatttaagcaaaaacatcgtagaaatacggtgtttttgttaccctaagtttaaac
tcctttttgataatctcatgaccaaaatcccttaacgtgagttttcgttccactgagcgtcagaccccgtagaaaagatcaaaggatcttcttgag
atcctttttttctgcgcgtaatctgctgcttgcaaacaaaaaaaccaccgctaccagcggtggtttgtttgccggatcaagagctaccaactctttt
tccgaaggtaactggcttcagcagagcgcagataccaaatactgttcttctagtgtagccgtagttaggccaccacttcaagaactctgtagca
ccgcctacatacctcgctctgctaatcctgttaccagtggctgctgccagtggcgataagtcgtgtcttaccgggttggactcaagacgatagt
taccggataaggcgcagcggtcgggctgaacggggggttcgtgcacacagcccagcttggagcgaacgacctacaccgaactgagata
cctacagcgtgagctatgagaaagcgccacgcttcccgaagggagaaaggcggacaggtatccggtaagcggcagggtcggaacagg
agagcgcacgagggagcttccagggggaaacgcctggtatctttatagtcctgtcgggtttcgccacctctgacttgagcgtcgatttttgtga
tgctcgtcaggggggcggagcctatggaaaaacgccagcaacgcggcctttttacggttcctggccttttgctggccttttgctcacatgttctt

tcctgcgttatcccctgattctgtggataaccgtattaccgcctttgagtgagctgataccgctcgccgcagccgaacgaccgagcgcagcga
gtcagtgagcgaggaagcggaagagcgcccaatacgcagggccccctgcttcggggtcattatagcgattttttcggtatatccatcctttttc
gcacgatatacaggattttgccaaagggttcgtgtagactttccttggtgtatccaacggcgtcagccgggcaggataggtgaagtaggccc
acccgcgagcgggtgttccttcttcactgtcccttattcgcacctggcggtgctcaacgggaatcctgctctgcgaggctggccggctaccgc
cggcgtaacagatgagggcaagcggatggctgatgaaaccaagccaaccaggaagggcagcccacctatcaaggtgtactgccttccag
acgaacgaagagcgattgaggaaaaggcggcggcggccggcatgagcctgtcggcctacctgctggccgtcggccagggctacaaaat
cacgggcgtcgtggactatgagcacgtccgcgagctggcccgcatcaatggcgacctgggccgcctgggcggcctgctgaaactctggc
tcaccgacgacccgcgcacggcgcggttcggtgatgccacgatcctcgccctgctggcgaagatcgaagagaagcaggacgagcttgg
caaggtcatgatgggcgtggtccgcccgagggcagagccatgactttttttagccgctaaaacggccgggggggtgcgcgtgattgccaagc
acgtccccatgcgctccatcaagaagagcgacttcgcggagctggtgaagtacatcaccgacgagcaaggcaagaccgatcgggccc

<u>pPthl_adhE2</u>: *adhE2* is displayed in orange and the P*thl* promoter in green.

cctgcaggataaaaaaattgtagataaattttataaaatagttttatctacaatttttttatcaggaaacagctatgaccgcggccgctgtatccatg
tcaagcaggagtaaaaagcgaaaagacctgtaaagaattgtggagtaaaggatacaaggtaattaatattaaagatggtacagattcgtaca
agcaaccacttgaaagataaaaaacaagagtaaaatgtaaaatagtctatgtgcttcatgaagctaatataatgaagcaaagactattttacatt
ctttattttttaaaatgattttatatagatatccttaagttcacttataagtggatacctaggattagctgttgtacattggtcatcaaaagcaagctctg
acattttatctagcgtattataaaaatctttttatttattccagcggcacttatattttgtggaatactcaaatctatctttaactttgaaatagcttctatt
aaggctgttaccttttcggtatcgctagtaccctttaaattcaaatactctgcaatttcagcatattttctcttagcattaggagatttatattgaggga
atgctgtttgctttgttggacagtctgtagcgttatatttaataacttcttctattaatacagcacaagcaattccatgtggaacgtgatgcattgccc
caagtttatgagccattgaatggcatacacctaagaaagcatttgcaaatgccatccccgcaatattagaggcatgtgccatttttctcttgcttc
aatgtcgttagtcccatttttataggctctaggcaaatatttaaatatcatttttattgctcttaaggctaattcatcagtataatccgtagccataacc
gaaacatatgcttctatagcatgaactaatgcatctattccagttgctgctgttaattttctaggcatatttaacattaattcagtatctattattgccat
gtttggggtcaattcataagaagttaaagggtatttcattcctgtttcatcattagttataactgcaaaaggtgttgcctctgaaccggtaccagca
gttgtaggaatagctactgaaatcgcctttgtacctaatttagggaaattgcatattctctttcttatatccataaagtttatagctagattttcaatttct

gcttctggatattcatataacaagtgcataacctttgctgcatccattggcgatccaccaccaatagagattatagtatcaggttcaaagttaagcatttctttagcacctttttttactgaatcaatagttggatcagatttaatatctgtaaatatactgtatttaatatctatctcatctagtacctttgttattttattaacatatccaagtttaaaaagatctttatctgttactataaaggctcttttcttattcatatcttttaattcttttaatgcaaatctaagacatccatatttaaaatatatttttgtggcactttaaaccaaagcatattttcccttctttcagcaacacttttaatatttaataaatgtttaggctctacattttgcgatacagagtttcctccccaagtgccgcatccaagagtaaatgatggtgctatcgcaaaattgtataaatctccgcttgctccctgtgaagaaggcatgttaataaatgtccttgaagttttcattgctaatccaaattctttaactttatccttattgttttgtgaatctatatataaagatgacgtgtgtccacttccacctaattctattagcctttgtgcctttttttagagcttcatcaaaatccttaactttatacattgcaagtactggtgatagtttttcatgtgagaacagctcgcttttttcaacagattgtacttcgcctataagtatctttgtagtttgaggaacttcaattcctgccattttagcaattatataagcagattttccaactatgtcagcattaatagctccatttttaaacatagtttcttttattttagctatttcattttgattgagtatatatgatcctcgttttacaaattcctctttaactttttcgtatattgaattcataactaatattgattgttcagaagcgcatattactccattgtcataagtctttgataaaattatggagcttactgccatatctatatctgcactctcatctattattgctggtgtatttcctgctccaacaccaattgcaggttttccagatgaataggccgctttaaccattgaaggacctcctgttgctaatattatatcagcttcactcatcaaatcttgagaaagttctattgatggctcatctatccagcctattatatttttaggtgctcctgctttaacagctgcatctaaaattaattttgctgcagcaattgtagatttttttgcacgtggatgtggtgaaaagaatattgcgtttcttgtttttaaagaaattaatgatttgaaaattgctgtggaagttggattagtagtaggaactatggctgcaacaattccaattggttcagcaacctttgttatgcctaaagaatcgtcatggtctattatgccacaagtttttttcatttttatatttattgtatatatattctgctgcaaaatgatttttttataattttatcttctacaagacctattcctgtttcttctactgctaatttagctaagtttattctttctttagctgcggctatggcacattgtttaaaaattttatcaacttgctcttgagtataggttgcaaacttcttttgcgcttctctcaattcatttagcttttgttttagttctttttgatttgtaactttcatatgaactaacctcctaaattttgatacggggtaacagataaaccatttcaatctatttcataagttccatagtttatccctaatttatacgttttctctaacaacttaattatacccactattattatttttatcaatatattttgttaaaaaataaaaataagaagcctgcatttgcaggcttcttatttttatggcgcgccgccattattttttgaacaattgacaattcatttcttattttttattaagtgatagtcaaaaggcataacagtgctgaatagaaagaaatttacagaaaagaaaattatagaatttagtatgattaattatactcatttatgaatgtttaattgaatacaaaaaaaaatacttgttatgtattcaattacgggttaaaatatagacaagttgaaaaatttaataaaaaaataagtcctcagctcttatatattaagctaccaacttagtatataagccaaaacttaaatgtgctaccaacacatcaagccgttagagaactctatctatagcaatatttcaaatgtaccgacatacaagagaaacattaactatatatattcaatttatgagattatcttaacagatataaatgtaaattgcaataagtaagatttagaagtttatagcctttgtgtattggaagcagtacgcaaaggctttttatttgataaaaattagaagtatatttatttttcataattaatttatgaaa

atgaaagggggtgagcaaagtgacagaggaaagcagtatcttatcaaataacaaggtattagcaatatcattattgactttagcagtaaacattatgacttttatagtgcttgtagctaagtagtacgaaagggggagctttaaaaagctccttggaatacatagaattcataaattaatttatgaaaagaagggcgtatatgaaaacttgtaaaaattgcaaagagtttattaaagatactgaaatatgcaaaatacattcgttgatgattcatgataaaacagtagcaacctattgcagtaaatacaatgagtcaagatgtttacataaagggaaagtccaatgtattaattgttcaaagatgaaccgatatggatggtgtgccataaaaatgagatgttttacagaggaagaacagaaaaaagaacgtacatgcattaaatattatgcaaggagctttaaaaaagctcatgtaaagaagagtaaaaagaaaaaataatttatttattaatttaatattgagagtgccgacacagtatgcactaaaaaatatatctgtggtgtagtgagccgatacaaaaggatagtcactcgcattttcataatacatcttatgttatgattatgtgtcggtgggacttcacgacgaaaacccacaataaaaaaagagttcggggtagggttaagcatagttgaggcaactaaacaatcaagctaggatatgcagtagcagaccgtaaggtcgttgtttaggtgtgttgtaatacatacgctattaagatgtaaaaatacggataccaatgaagggaaaagtataatttttggatgtagtttgtttgttcatctatgggcaaactacgtccaaagccgtttccaaatctgctaaaaagtatatcctttctaaaatcaaagtcaagtatgaaatcataaataaagtttaattttgaagttattatgatattatgtttttctattaaaataaattaagtatatagaatagtttaataatagtatatacttaatgtgataagtgtctgacagtgtcacagaaaggatgattgttatggattataagcggccggccagtgggcaagttgaaaaattcacaaaaatgtggtataatatctttgttcattagagcgataaacttgaatttgagagggaacttagatggtatttgaaaaaattgataaaaatagttggaacagaaaagagtattttgaccactactttgcaagtgtaccttgtacctacagcatgaccgttaaagtggatatcacacaaataaaggaaaagggaatgaaactatatcctgcaatgctttattatattgcaatgattgtaaaccgccattcagagtttaggacggcaatcaatcaagatggtgaattggggatatatgatgagatgataccaagctatacaatatttcacaatgatactgaaacattttccagcctttggactgagtgtaagtctgactttaaatcatttttagcagattatgaaagtgatacgcaacggtatggaaacaatcatagaatggaaggaaagccaaatgctccggaaaacatttttaatgtatctatgataccgtggtcaaccttcgatggctttaatctgaatttgcagaaaggatatgattatttgattcctatttttactatggggaaatattataaagaagataacaaaattatacttcctttggcaattcaagttcatcacgcagtatgtgacggatttcacatttgccgttttgtaaacgaattgcaggaattgataaatagttaacttcaggtttgtctgtaactaaaaacaagtatttaagcaaaaacatcgtagaaatacggtgtttttgttaccctaagtttaaactcctttttgataatctcatgaccaaaatcccttaacgtgagttttcgttccactgagcgtcagaccccgtagaaaagatcaaaggatcttcttgagatcctttttttctgcgcgtaatctgctgcttgcaaacaaaaaaaccaccgctaccagcggtggtttgtttgccggatcaagagctaccaactctttttccgaaggtaactggcttcagcagagcgcagataccaaatactgttcttctagtgtagccgtagttaggccaccacttcaagaactctgtagcaccgcctacatacctcgctctgctaatcctgttaccagtggctgctgccagtggcgataagtcgtgtcttaccgggttggactcaagacgatagttaccggataaggcgcagcggtcgggctgaacggggggttc

gtgcacacagcccagcttggagcgaacgacctacaccgaactgagatacctacagcgtgagctatgagaaagcgccacgcttcccgaag
ggagaaaggcggacaggtatccggtaagcggcagggtcggaacaggagagcgcacgagggagcttccagggggaaacgcctggtat
ctttatagtcctgtcgggtttcgccacctctgacttgagcgtcgatttttgtgatgctcgtcaggggggcggagcctatggaaaaacgccagca
acgcggcctttttacggttcctggccttttgctggccttttgctcacatgttctttcctgcgttatcccctgattctgtggataaccgtattaccgcctt
tgagtgagctgataccgctcgccgcagccgaacgaccgagcgcagcgagtcagtgagcgaggaagcggaagagcgcccaatacgcag
ggccccctgcttcggggtcattatagcgatttttcggtatatccatcctttttcgcacgatatacaggattttgccaaagggttcgtgtagactttc
cttggtgtatccaacggcgtcagccgggcaggataggtgaagtaggcccacccgcgagcgggtgttccttcttcactgtcccttattcgcacc
tggcggtgctcaacgggaatcctgctctgcgaggctggccggctaccgccggcgtaacagatgagggcaagcggatggctgatgaaacc
aagccaaccaggaagggcagcccacctatcaaggtgtactgccttccagacgaacgaagagcgattgaggaaaaggcggcggcggcc
ggcatgagcctgtcggcctacctgctggccgtcggccagggctacaaaatcacgggcgtcgtggactatgagcacgtccgcgagctggc
ccgcatcaatggcgacctgggccgcctgggcggcctgctgaaactctggctcaccgacgacccgcgcacggcgcggttcggtgatgcca
cgatcctcgccctgctggcgaagatcgaagagaagcaggacgagcttggcaaggtcatgatgggcgtggtccgcccgagggcagagcc
atgactttttagccgctaaaacggccggggggtgcgcgtgattgccaagcacgtccccatgcgctccatcaagaagagcgacttcgcgga
gctggtgaagtacatcaccgacgagcaaggcaagaccgatcgggccc

<u>pPthl_adhE2_bdhB</u>: *bdhB* is shown in gray, *adhE2* in orange and the P*thl* promoter in green.

cctgcaggataaaaaaattgtagataaattttataaaatagttttatctacaatttttttatactccccaagatattaatgctttgggggggtatttttatt
tatagttgaatgtctaaattttctctctgctttgagttttatttctttgctagcatactgctttcttggagttcatgaatataatggctacgcccaaaccc
cggtaggtttacacagatttttgaatatttgtaggacttcggaggcgtttactggtcttaggtttcctatggttcctcctgtaagctttactgattcctt
tgccattatgtccaatttttcttcttcaattccaacatctctcagtctagatggtaaacctagtacatttacaaagtaatctcttgtttttttgtattgcttga
tgtgctatgtcatagtgatttttttctttgtctattccccaaacatttacaccatattcaacaaacttgtacactgtatcattatttaaaatatactccatc
caattaggtgttaaaattgcaagccctacgccgtgtgttatgtcgtaataagcacttaattcatgttccattaagtgtacactccaattagtgtcttta
ccatatgttaaaagtccatttatcgcaagacttgaagcccacattagattggctcttgcctcataatcatccggcttctcaagagctattcctccat

atttaatacaagttcttaataacgcttctgccattctatcctgcaaatatgctgtttttgtattactaaaatacacctcaaatatatgactcataatatcagctgttcctgctgctgtttgattggtaggtacggtatacgtatacgttggatctaatatagaaaacttaggagccatatctggatgtgccgcaattagtttttcgtttgtatccatattatttattactgcccacgtatccatttctgatcctgttgcagcaatggttaatatactagctataggaagcacccttttatttttgagccatctaacacaatatcccatggatttccatcatattcacatgctgctgctataacctttgcgcaatctattgcacttcctccacctatagctagtactacttcaactccattttctctacatattttaactcctttttcaactgtagttactcttggatttggctctactcctgcaagttcataaaatttaatactgtttttttcaagtatacttacagctttatcatatattccatttctctttatacttcctccaccataaactataagcactttagaaccatattttttaagctctcttccaagtacatttatcttatctttaccgaaaaaaattctagttggtattgaatattcgaaatcaaccactattttttaaaatgattttatatagatatccttaagttcacttataagtggatacctaggattagctgttgtacattggtcatcaaaagcaagctctgacattttatctagcgtattataaaaatcttttttatttattccagcggcacttatattttgtggaatactcaaatctatctttaactttgaaatagcttctattaaggctgttaccttttcggtatcgctagtaccctttaaattcaaatactctgcaatttcagcatattttctcttagcattaggagatttatattgagggaatgctgtttgctttgttggacagtctgtagcgttatatttaataacttcttctattaatacagcacaagcaattccatgtggaacgtgatgcattgccccaagtttatgagccattgaatggcatacacctaagaaagcatttgcaaatgccatccccgcaatattagaggcatgtgccatttttttctcttgcttcaatgtcgttagtcccatttttataggctctaggcaaatatttaaatatcatttttattgctcttaaggctaattcatcagtataatccgtagccataaccgaaacatatgcttctatagcatgaactaatgcatctattccagttgctgctgttaattttctaggcatatttaacattaattcagtatctattattgccatgtttggggtcaattcataagaagttaaagggtatttcattcctgtttcatcattagttataactgcaaaaggtgttgcctctgaaccggtaccagcagttgtaggaatagctactgaaatcgcctttgtacctaatttagggaaattgcatattctctttcttatatccataaagtttatagctagattttcaatttctgcttctggatattcatataacaagtgcataacctttgctgcatccattggcgatccaccaccaatagagattatagtatcaggttcaaagttaagcatttctttagcaccttttttttactgaatcaatagttggatcagatttaatatctgtaaatatactgtatttaatatctatctcatctagtacctttgttattttattaacatatccaagtttaaaaagatctttatctgttactataaaggctcttttcttattcatatcttttaattcttttaatgcaaatctaagacatccatatttaaaatatattttttgtggcactttaaaccaaagcatattttcccttctttcagcaacacttttaatatttaataaatgtttaggctctacattttgcgatacagagtttcctccccaagtgccgcatccaagagtaaatgatggtgctatcgcaaaattgtataaatctccgcttgctccctgtgaagaaggcatgttaataaatgtccttgaagttttcattgctaatccaaattctttaactttatccttattgttttgtgaatctatatataaagatgacgtgtgtccacttccacctaattctattagcctttgtgccttttttagagcttcatcaaaatccttaactttatacattgcaagtactggtgatagtttttcatgtgagaacagctcgctttttttcaacagattgtacttcgcctataagtatctttgtagtttgaggaacttcaattcctgccattttagcaattatataagcagattttccaactatgtcagcattaatagctccatttttaaac

atagtttcttttattttagctatttcattttgattgagtatatatgatcctcgttttacaaattcctctttaactttttcgtatattgaattcataactaatattga
ttgttcagaagcgcatattactccattgtcataagtctttgataaaattatggagcttactgccatatctatatctgcactctcatctattattgctggt
gtatttcctgctccaacaccaattgcaggttttccagatgaataggccgctttaaccattgaaggacctcctgttgctaatattatatcagcttcact
catcaaatcttgagaaagttctattgatggctcatctatccagcctattatatttttaggtgctcctgctttaacagctgcatctaaaattaattttgct
gcagcaattgtagatttttttgcacgtggatgtggtgaaaagaatattgcgtttcttgtttttaaagaaattaatgatttgaaaattgctgtggaagtt
ggattagtagtaggaactatggctgcaacaattccaattggttcagcaacctttgttatgcctaaagaatcgtcatggtctattatgccacaagttt
tttcatttttatatttattgtatatatattctgctgcaaaatgatttttataattttatcttctacaagacctattcctgtttcttctactgctaatttagctaag
tttattctttctttagctgcggctatggcacattgtttaaaaattttatcaacttgctcttgagtataggttgcaaacttcttttgcgcttctctcaattcat
ttagcttttgttttagttctttttgatttgtaactttcatatgaactaacctcctaaattttgatacggggtaacagataaaccatttcaatctatttcataa
gttccatagtttatccctaatttatacgttttctctaacaacttaattatacccactattattatttttatcaatatattttgttaaaaaataaaaataagaa
gcctgcatttgcaggcttcttatttttatggcgcgccgccattattttttgaacaattgacaattcatttcttatttttttattaagtgatagtcaaaaggc
ataacagtgctgaatagaaagaaatttacagaaaagaaaattatagaatttagtatgattaattatactcatttatgaatgtttaattgaatacaaaa
aaaaatacttgttatgtattcaattacgggttaaaatatagacaagttgaaaaatttaataaaaaaataagtcctcagctcttatatattaagctacc
aacttagtatataagccaaaacttaaatgtgctaccaacacatcaagccgttagagaactctatctatagcaatatttcaaatgtaccgacataca
agagaaacattaactatatatattcaatttatgagattatcttaacagatataaatgtaaattgcaataagtaagatttagaagtttatagcctttgtgt
attggaagcagtacgcaaaggctttttttatttgataaaaattagaagtatatttattttttcataattaatttatgaaaatgaaaggggggtgagcaaa
gtgacagaggaaagcagtatcttatcaaataacaaggtattagcaatatcattattgactttagcagtaaacattatgacttttatagtgcttgtag
ctaagtagtacgaaagggggagctttaaaaagctccttggaatacatagaattcataaattaatttatgaaaagaagggcgtatatgaaaactt
gtaaaaattgcaaagagtttattaaagatactgaaatatgcaaaatacattcgttgatgattcatgataaaacagtagcaacctattgcagtaaat
acaatgagtcaagatgtttacataaagggaaagtccaatgtattaattgttcaaagatgaaccgatatggatggtgtgccataaaaatgagatgt
tttacagaggaagaacagaaaaaagaacgtacatgcattaaatattatgcaaggagctttaaaaaagctcatgtaaagaagagtaaaaagaa
aaaataatttatttattaatttaatattgagagtgccgacacagtatgcactaaaaaatatatctgtggtgtagtgagccgatacaaaaggatagtc
actcgcattttcataatacatcttatgttatgattatgtgtcggtgggacttcacgacgaaaacccacaataaaaaaagagttcggggtagggtta
agcatagttgaggcaactaaacaatcaagctaggatatgcagtagcagaccgtaaggtcgttgtttaggtgtgttgtaatacatacgctattaa

gatgtaaaaatacggataccaatgaagggaaaagtataatttttggatgtagtttgtttgttcatctatgggcaaactacgtccaaagccgtttcc
aaatctgctaaaaagtatatcctttctaaaatcaaagtcaagtatgaaatcataaataaagtttaattttgaagttattatgatattatgtttttctattaa
aataaattaagtatatagaatagtttaataatagtatatacttaatgtgataagtgtctgacagtgtcacagaaaggatgattgttatggattataag
cggccggccagtgggcaagttgaaaaattcacaaaaatgtggtataatatctttgttcattagagcgataaacttgaatttgagagggaactta
gatggtatttgaaaaaattgataaaaatagttggaacagaaaagagtattttgaccactactttgcaagtgtaccttgtacctacagcatgaccgt
taaagtggatatcacacaaataaaggaaaagggaatgaaactatatcctgcaatgctttattatattgcaatgattgtaaaccgccattcagagtt
taggacggcaatcaatcaagatggtgaattggggatatatgatgagatgataccaagctatacaatatttcacaatgatactgaaacattttcca
gcctttggactgagtgtaagtctgactttaaatcatttttagcagattatgaaagtgatacgcaacggtatggaaacaatcatagaatggaagga
aagccaaatgctccggaaaacatttttaatgtatctatgataccgtggtcaaccttcgatggctttaatctgaatttgcagaaaggatatgattattt
gattcctatttttactatggggaaatattataaagaagataacaaaattatacttcctttggcaattcaagttcatcacgcagtatgtgacggatttc
acatttgccgttttgtaaacgaattgcaggaattgataaatagttaacttcaggtttgtctgtaactaaaaacaagtatttaagcaaaaacatcgta
gaaatacggtgtttttgttaccctaagtttaaactcctttttgataatctcatgaccaaaatcccttaacgtgagttttcgttccactgagcgtcaga
ccccgtagaaaagatcaaaggatcttcttgagatcctttttttctgcgcgtaatctgctgcttgcaaacaaaaaaaccaccgctaccagcggtg
gtttgtttgccggatcaagagctaccaactctttttccgaaggtaactggcttcagcagagcgcagataccaaatactgttcttctagtgtagcc
gtagttaggccaccacttcaagaactctgtagcaccgcctacatacctcgctctgctaatcctgttaccagtggctgctgccagtggcgataag
tcgtgtcttaccgggttggactcaagacgatagttaccggataaggcgcagcggtcgggctgaacggggggttcgtgcacacagcccagc
ttggagcgaacgacctacaccgaactgagatacctacagcgtgagctatgagaaagcgccacgcttcccgaagggagaaaggcggaca
ggtatccggtaagcggcagggtcggaacaggagagcgcacgagggagcttccagggggaaacgcctggtatctttatagtcctgtcgggt
ttcgccacctctgacttgagcgtcgatttttgtgatgctcgtcaggggggcggagcctatggaaaaacgccagcaacgcggcctttttacggtt
cctggccttttgctggccttttgctcacatgttctttcctgcgttatcccctgattctgtggataaccgtattaccgcctttgagtgagctgataccgc
tcgccgcagccgaacgaccgagcgcagcgagtcagtgagcgaggaagcggaagagcgcccaatacgcagggccccctgcttcggggt
cattatagcgatttttttcggtatatccatcctttttcgcacgatatacaggattttgccaaagggttcgtgtagactttccttggtgtatccaacggcg
tcagccgggcaggataggtgaagtaggcccacccgcgagcgggtgttccttcttcactgtcccttattcgcacctggcggtgctcaacggga
atcctgctctgcgaggctggccggctaccgccggcgtaacagatgagggcaagcggatggctgatgaaaccaagccaaccaggaaggg

cagcccacctatcaaggtgtactgccttccagacgaacgaagagcgattgaggaaaaggcggcggcggccggcatgagcctgtcggcct
acctgctggccgtcggccagggctacaaaatcacgggcgtcgtggactatgagcacgtccgcgagctggcccgcatcaatggcgacctg
ggccgcctgggcggcctgctgaaactctggctcaccgacgacccgcgcacggcgcggttcggtgatgccacgatcctcgccctgctggc
gaagatcgaagagaagcaggacgagcttggcaaggtcatgatgggcgtggtccgcccgagggcagagccatgacttttttagccgctaaa
acggccggggggtgcgcgtgattgccaagcacgtccccatgcgctccatcaagaagagcgacttcgcggagctggtgaagtacatcacc
gacgagcaaggcaagaccgatcgggccc

References:

1. Fink C, Beblawy S, Enkerlin AM, Mühling L, Angenent LT, Molitor B. 2021. A shuttle-vector system allows heterologous gene expression in the thermophilic methanogen *Methanothermobacter thermautotrophicus* ΔH. MBio 12:e02766-21.
2. Xia P-F, Casini I, Schulz S, Klask C-M, Angenent LT, Molitor B. 2020. Reprogramming acetogenic bacteria with CRISPR-targeted base editing *via* deamination. ACS Synthetic Biology 9:2162-2171.
3. Molitor B, Kirchner K, Henrich AW, Schmitz S, Rosenbaum MA. 2016. Expanding the molecular toolkit for the homoacetogen *Clostridium ljungdahlii.* Scientific Reports 6:1-10.
4. Klask C-M, Jäger B, Casini I, Angenent LT, Molitor B. 2022. Genetic evidence reveals the indispensable role of the *rseC* gene for autotrophy and the importance of a functional electron balance for nitrate reduction in *Clostridium ljungdahlii*. Frontiers in Microbiology 13:887578.
